# Tunable bioactivity and mechanics of collagen-based tissue engineering constructs: A comparison of EDC-NHS, genipin and TG2 crosslinkers

**DOI:** 10.1016/j.biomaterials.2020.120109

**Published:** 2020-09

**Authors:** Malavika Nair, Ramneek K. Johal, Samir W. Hamaia, Serena M. Best, Ruth E. Cameron

**Affiliations:** a Cambridge Centre for Medical Materials, Department of Materials Science and Metallurgy, University of Cambridge, 27 Charles Babbage Road, Cambridge, CB3 0FS, United Kingdom; b Department of Biochemistry, University of Cambridge, 8 Tennis Court Road, Cambridge, CB2 1QW, United Kingdom

**Keywords:** Collagen, Crosslinking, EDC-NHS, Genipin, TG2

## Abstract

Due to its ubiquity and versatility in the human body, collagen is an ideal base material for tissue-engineering constructs. Chemical crosslinking treatments allow precise control of the biochemical and mechanical properties through macromolecular modifications to the structure of collagen. In this work, three key facets regarding the collagen crosslinking process are explored. Firstly, a comparison is drawn between the carbodiimide-succinimide (EDC-NHS) system and two emerging crosslinkers utilising alternate chemistries: genipin and tissue transglutaminase (TG2). By characterising the chemical changes upon treatment, the effect of EDC-NHS, genipin and TG2 crosslinking mechanisms on the chemical structure of collagen, and thus the mechanical properties conferred to the substrate is explored. Secondly, the relative importance of mechanical and biochemical cues on cellular phenomena are investigated, including cell viability, integrin-specific attachment, spreading and proliferation. Here, we observe that for human dermal fibroblasts, long-term, stable proliferation is preconditioned by the availability of suitable binding sites, irrespective of the substrate modulus post-crosslinking. Finally, as seen in the graphical abstract we show that by choosing the appropriate crosslinker chemistries, a materials selection map can be drawn for collagen films, encompassing both a range of tensile modulus and fibroblast proliferation which can be modified independently. Thus, in addition to a range of parameters that can be modified in collagen constructs, we demonstrate a route to obtaining tunable bioactivity and mechanics in collagen constructs is uncovered, that is exclusively driven by the crosslinking process.

## Graphical abstract

**Figure:**
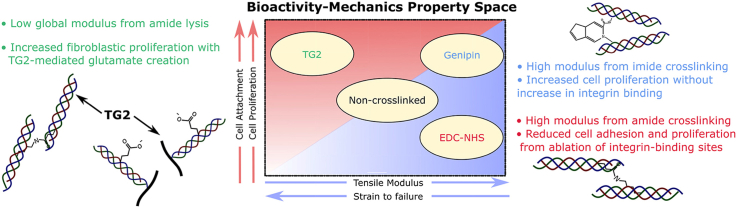

## 1 Introduction

Collagen-I, a primary protein component of the extracellular matrix (ECM) has been widely employed as a base material for tissue engineering constructs with the aim of providing a similar chemical environment to the one experienced *in vivo* [[1], [2], [3]]. The ubiquity of collagen in connective tissue has promoted its use in medical devices tailored for a broad range of applications. These include injectable hydrogels for cartilage repair [4] and dense fibrillar matrices prepared from neutralised acid-soluble collagen for dermal wound healing [5], collagen membranes as a periodontal barrier [6] and freeze-dried porous scaffolds fabricated from insoluble microfibrillar collagen for ex-vivo platelet production in flow bioreactors [7]. Though they vary in the type, source or solubility of collagen, these constructs can be crosslinked to tune their mechanical properties to match the loads experienced in-service at the host tissue site. Collagen-I fibres naturally possess amide crosslinks along their polypeptide chains, providing mechanical [[8], [9], [10]] and proteolytic [[11], [12], [13]] stability to the fibres. To mimic these natural crosslinks, amine-based crosslinkers [[14], [15], [16], [17], [18]] have been considered to be ideal candidates to crosslink processed collagen in tissue engineering constructs.

Collagen is recognised by a range of cell receptors including integrins, discoidin domain receptors, LAIR-1, mannose receptors and glycoprotein VI, of which integrin receptors comprise the primary group [19]. These receptors are heterodimers composed of an and subunit [20]. Collagen specifically binds to the metal ion-dependent adhesion site (the MIDAS motif) in the integrins. This metal ion, often magnesium, is stabilised by glutamates (E), and results in a specificity for GXOGER motifs expressed by the tropocollagen molecules within the collagen fibres [19].

One of the most common crosslinkers for collagen includes a combination of 1-ethyl-3(3-dimethylaminopropyl-carbodiimide hydrochloride (EDC) with N-hydroxysuccinimide (NHS), with early work by Olde Damink et al. [15] establishing industry standards for the crosslinker ratios to be used with collagen. However, despite its popularity, EDC-NHS has been shown to produce low levels of integrin-specific cell binding at higher crosslinking conditions [21]. This phenomenon has been purported to be caused by the interaction between EDC and the carboxylate anions (E) within the MIDAS motifs in collagen (Supplementary Fig. 1a). It has also been suggested that lost cell adhesion can be subsequently restored through the artificial introduction of the peptide sequence post-crosslinking [22].

Genipin and tissue transglutaminase (TG2) present two alternative amine based crosslinkers that have been widely used with tissue engineering constructs: genipin is commonly used with chitosan [23] whereas TG2 has found use with soluble collagen [17] and other proteins of the extracellular matrix including thrombin, fibrin and gelatin [24]. Genipin crosslinks collagen via an imide crosslinking mechanism (Supplementary Fig. 1b), whereby primary lysines in the collagen backbone are bound to genipin precursor [25]. Genipin therefore does not crosslink via the glutamic acids (E) in collagen, and leaves the GXOGER motifs unaffected during the crosslinking process.

Transglutaminases are a family of transferases responsible for crosslinking of skin, hair and blood clots *in vivo* and have shown exceptional cellular responses, including increased attachment of human osteoblasts to collagen scaffolds upon crosslinking [26]. Transglutaminases bind to glutamines in the polypeptide chain, activating them for further reactions (Supplementary Fig. 1c) [27]. In the presence of water, this results in the conversion of the glutamine to a glutamate, whereas in the presence of a suitable amine, an amide bond is formed at the site of the activated glutamine [27]. Thus TG2 can either serve to act as an amide crosslinker that does not utilise pre-existing aspartic or glutamic acids (E and D) in the formation of crosslinks, or conversely could help re-introduce glutamates (E) in the substrate, thus increasing the number of MIDAS motifs on the substrate.

However, treating collagen with any crosslinker can alter the structure of collagen I across various length scales. Additionally, the wide variety of formats adopted by collagen-based medical devices gives rise to a range of two–three dimensional architectures that can be fabricated, which can also contribute to differences in cellular behaviour. The concomitant alteration of various biochemical and structural cues upon crosslinking creates great difficulty in attributing cell responses to a single property of the substrate. Two dimensional films of insoluble microfibrillar collagen-I can be used in place of neutralised collagen monolayer coatings or three-dimensional scaffolds as a surrogate in the assessment of cellular response without introducing the complexity of microarchitecture, macroscopic swelling and fluid flow that can be varied in three dimensional architecture [28]. Using human dermal fibroblasts (HDFs) as a cheap and robust cell line expressing a stable phenotype and a range of integrins that can bind to collagen [29] also allows for the comparison of integrin-specific cell attachment to longer term cell proliferation and viability. Through rigorous consideration of the mechanochemical influence of the crosslinkers on collagen as well as the cellular interactions with the treated films, further insights are provided here to uncouple the effects of mechanical and chemical cues on cellular response.

## 2 Materials and Methods

### 2.1 Film fabrication

Type I dermal microfibrillar bovine collagen (18–36 month *Bos Taurus* cattle, 38.8 mmol amide N/100g, 76.1 ± 1.6% total protein content not including Cys and Trp). A 0.5% suspension of collagen-I was prepared in 0.05 M acetic acid solution (Alfa Aesar) and left to swell overnight at 4 °C. Suspensions were homogenised at 22,000 rpm for 4 min in total using a commercial blender (Waring model 8011 EG). The resulting suspension was degassed under vacuum to remove any air bubbles. Films were prepared by adding 400 l of suspension to a 24 well plate (Corning) or 200 ml to a 48 well plate (Corning). Films were left to dry for 48 h before crosslinking.

### 2.2 Crosslinking

#### 2.2.1 EDC-NHS

A crosslinking solution of 1-ethyl-3-(3-dimethylaminopropyl) carbodiimide hydrochloride (EDC, Sigma-Aldrich) and N-hydroxysuccinimide (NHS, Sigma-Aldrich) was prepared using a 5:2:1 M ratio for EDC: NHS:Collagen, hereafter referred to as the ‘100% concentration’. For every 1 g of collagen, the 100% concentration standard of crosslinking solution consisted of 1.15 g EDC and 0.276 g NHS dissolved in 75% ethanol. Diluted crosslinking solutions were prepared at a molar ratio of 5:2:2 and 5:2:10 EDC:NHS:Collagen, hereby referred to as the ‘50%’ and ‘10%’ crosslinking concentrations for EDC-NHS. Films were immersed in 100%, 50% and 10% crosslinking solution for 2 h at ambient temperature.

#### 2.2.2 Genipin

Using a mass for mass equivalence for genipin to PBS adapted from the protocol by Zhang et al. [30], for every 1 g of collagen the 100% concentration standard of crosslinking solution consisted of 0.7812 g of genipin (Challenge Bioproducts 160919) dissolved in PBS (Sigma Aldrich). Crosslinking solutions prepared at a mass ratio of 1.5624 g of genipin per gram of collagen are referred to as the ‘200%’ and mass ratio of 0.46872 g genipin per gram of collagen referred to as the ‘60%’ crosslinking concentrations for genipin. Samples were immersed in 60%, 100% and 200% crosslinking solution for 24 h at ambient temperature.

#### 2.2.3 Tissue transglutaminase 2

Using a mass for mass equivalence for TG2 (from guinea pig liver, 99% purity, 4.8 units/mg protein, Sigma Aldrich T5398) and Tris buffer in the protocol adopted by Chau et al. [26], for every 1 g of collagen the 100% concentration standard of crosslinking solution consisted of 1 mg of TG2 (Sigma Aldrich) dissolved in Tris Buffer pH 7.5 (Sigma Aldrich) and 5 mM CaCl2 (Sigma Aldrich). Solutions prepared with 0.4 mg of TG2 per gram of collagen and 2.0 mg of TG2 per gram of collagen are referred to as the ‘40%’ and ‘200%’ crosslinking concentrations for TG2. Samples were immersed in 40%, 100% and 200% crosslinking solutions for 24 h at 37 °C.

All crosslinked films were washed with distilled water (10 s × 4) followed by soaking in distilled water for longer intervals (15 min × 4). Films were left to dry for 48 h before use.

### 2.3 Free amine ninhydrin assay

The free amine content of collagen films was determined using the ninhydrin assay. A solution of ninhydrin was prepared by adding 0.25 g of ninhydrin to 5 ml of ethanol per mg of collagen. Ninhydrin solution was added to each well of collagen crosslinked films, then plates were sealed with Parafilm and heated to 80 °C for 20 min. Plates were cooled to ambient room temperature and eluted with 50% isopropanol (1.25 ml isopropanol per 1 ml of ninhydrin solution). 200 l of the resultant Ruhemann's purple dye was transferred to a 96 well plate and the absorbance was read at 570 nm using a using a SPECTROstar Nano plate reader (BMG LABTECH Ltd, Aylesbury, UK). A standard curve was prepared using glycine (Sigma-Aldrich) and the linear region of absorbance with concentration was used to convert sample absorbances to amine content. The degree of crosslinking was then determined according to the formula:

$$\text{Degree of crosslinking }\left(\text{\%}\right)=\left(1-\frac{\text{Amine content in sample}}{\text{Amine content in non}-\text{crosslinked}}\right)\times 100\text{\%}$$

### 2.4 FTIR

Fourier-transform Infrared (FTIR) spectra of the free standing collagen treated and untreated films were obtained in ATR mode, with a wavenumber resolution of 4 ${\text{cm}}^{-1}$. The amide I peaks were subsequently deconvolved, and the proportion of the total amide peak area occupied by the triple helical peak was then obtained through the Peak Analyzer tool in Origin (Maximum Constant Baseline Subtraction, Hidden peak finding by second derivative). Data are presented as the mean percentage of the area occupied by the triple helical component and -helical components ± standard deviation of three replicates.

### 2.5 Tensile testing

Collagen films were cast and cut into 35 mm × 15 mm rectangular strips, then crosslinked with EDC-NHS, genipin and TG2. The dimensions of each film were measured post crosslinking, and an average thickness for each film was obtained across 10 measurements from optical micrographs. Mechanical testing of the films was then carried out using a 5 N load cell in a hydrated chamber in Hounsfield tensile tester (Tinius Olsen) at a constant extension rate of 5 mm ${\text{min}}^{-1}$. All samples were stretched until failure or yielding and data were used to plot a stress–strain curve. The tensile modulus (E) was defined as the gradient at the initial linear portion of the stress-strain. Results are reported as means ± standard deviations of an experiment performed in triplicate.

### 2.6 Cell culture

Human dermal fibroblasts were purchased from the European Collection of Animal Cell Cultures (Porton Down, UK) and maintained in a humidified incubator at 37 °C with 5% ${\text{CO}}_{2}$. Cells were cultured in Dulbecco's Modified Eagle Medium (DMEM) (Sigma-Aldrich) with 10% foetal bovine serum and 1% streptavidin/penicillin. When needed, cells were detached from cell culture flasks with 0.05% Trypsin/0.02% EDTA (Sigma-Aldrich) and re-suspended in serum free DMEM.

### 2.7 LDH cell attachment assay

Crosslinked films prepared in 48 well plates (Corning) were pre-blocked with 200 l 5% (w/v) bovine serum albumin (Sigma-Aldrich) for 1 h at ambient room temperature. Wells were washed with 200 l of PBS (Invitrogen) and 200 l of human dermal fibroblasts (1$\times 10^{5}$ cells/ml) suspended in serum free DMEM media containing either 5 mM MgCl^2^ or 5 mM EDTA were added for 1 h at 37 °C. Plates were washed three times with PBS to remove loosely bound cells (200 l) and permeabilised with 100 l per well of 2% Triton-X lysis buffer (in water) for 90 min at ambient room temperature. Subsequently, 100 l of lactate dehydrogenase (LDH) substrate (Cytotoxicity detection kit, Roche), prepared according to the manufacturer's instructions was added per well for 20 min and the absorbance read at 490 nm using a SPECTROstar Nano plate reader (BMG LABTECH Ltd, Aylesbury, UK). Results are reported as means ± standard deviations of three independent experiments performed in triplicate.

### 2.8 MTS cell proliferation assay

Crosslinked films prepared in 24 well plates (Corning) were seeded with human dermal fibroblasts ($5\times 10^{4}$) in phenol red free DMEM (Invitrogen) with 10% FBS and 1% penicillin/streptomycin for 1, 3 and 7 days and placed in a 37 °C incubator with 5% ${\text{CO}}_{2}$. Media was changed every 2 days and frozen for cytotoxicity assays. At each time point, MTS solution (Promega, CellTiter 96; 20 l per 100 l of media) was added and the plate incubated for 2 h at 37 °C. 100 l of solution was transferred to a 96 well plate (Corning) and the absorbance read at 490 nm using a SPECTROstar Nano plate reader (BMG LABTECH Ltd, Aylesbury, UK). A standard curve with known cell numbers was used to convert absorbance readings to cell number. Results are reported as means ± standard deviations of three independent experiment performed in triplicate.

### 2.9 LDH cell death assay

Spent media collected from the cell proliferation assay was spun down at 300 rpm to remove any cells and 100 l pipetted into a 96 well-plate (Corning). 100 l of LDH substrate (Roche) was added and colour developed for 20 min at 37 °C. The absorbance was read at 490 nm using a SPECTROstar Nano plate reader (BMG LABTECH Ltd, Aylesbury, UK).

### 2.10 Live-dead cell imaging

The viability of fibroblasts seeded on crosslinked collagen films on days 1, 4 and 7 was assessed using the LIVE/DEAD assay (Invitrogen). Wells were washed with PBS (×3) and stained with 4 M calcein-AM (LIVE) and 2 M ethidium homodimer-1 (DEAD) for 40 min at ambient room temperature. Images were taken using a Zeiss Axio Observer Z1 microscope with an AxioCam 503 camera (10× objective lens; Carl Zeiss Ltd, Cambridge, UK). The calcein-AM dye was detected using excitation/emission set at 495 nm/515 nm and the nucleus staining ethidium homodimer-1 dye detected at excitation/emission 495 nm/635 nm. Brightness and contrast of each channel was then adjusted prior to merging the image channels to minimise signal from autofluorescent collagen fibres in the background.

### 2.11 Cell spreading imaging

#### 2.11.1 Immunofluorescent staining

Crosslinked collagen plates (24 well) were pre-blocked with 800 l of 5% BSA (w/v; Sigma Aldrich) for 60 min at room temperature and seeded with 5000 cells suspended in serum free DMEM media for 2 h at 37 °C. The plates were subsequently washed with PBS (×3) and fixed with 3% glutaraldehyde (Sigma-Aldrich) for 20 min at room temperature. Plates were washed with PBS and permeabilised with 0.5% Triton-X (Sigma-Aldrich) for 5 min. Cells were stained with Flash Phalloidin-594 (Biolegend) for 40 min at room temperature, followed by staining with DAPI (Sigma-Aldrich) for 2 min in water. Plates were imaged using a Zeiss Axio Observer Z1 microscope with an AxioCam 503 camera (20× and 40× objective lens; Carl Zeiss Ltd, Cambridge, UK).

#### 2.11.2 Image processing

Images were subsequently processed and analysed using the Fiji image processing package of ImageJ (NIH). A 100 pixel rolling ball radius background subtraction was applied to the DAPI channel (350 nm excitation/450 nm emission) and a 300 pixel rolling ball radius to the phalloidin channel (590 nm excitation/611 nm emission). Brightness and contrast of each channel was then adjusted prior to merging the image channels to minimise signal from autofluorescent collagen fibres. Single cells were chosen and isolated using the colour threshold in the phalloidin channel.

### 2.12 Collagen-I expression

Crosslinked films prepared in 24 well plates (Corning) were seeded with human dermal fibroblasts ($5\times 10^{4}$) in phenol red free DMEM (Invitrogen) with 10% FBS and 1% penicillin/streptomycin and placed in a 37 °C incubator with 5% ${\text{CO}}_{2}$ for 7 days, with media replacement on Day 3. Media from the samples were extracted on Day 7 for an MMP1 ELISA analysis.

Total RNA was extracted from human dermal fibroblasts using the Mini Kit (Qiagen) and subjected to reverse transcription (using random hexamers) followed by quantitative polymerase chain reaction (PCR). The human Collagen-I alpha 1 gene on Chromosome 17 from exon 1 to exon 49 was reannotated (included as part of the Open Data). Collagen type I alpha chain forward and reverse primers were designed as 5′ CTGGCAGCAAAGGAGACAC 3’ (exon 20) and 5′ TCGCTTTCCTTCCTCTCCAG 3’ (exon 21), respectively. A TaqMan fluorogenic probe (5′-6FAM- ACCAACAGGGCCAGGCTCTCCC 3′) was designed for Col-I alpha chain quantification, binding to the 3′ end of exon 20 and 5′ end of exon 21. The probe contains a fluorescent reporter 6-carboxyfluorescein (FAM) at the 5′ end and a fluorescent quencher 6-carboxytetramethylrhodamine (TAMRA) at the 3′ end. Real-time quantitative PCR was performed on the cDNA of both the Collagen-I alpha chain and as an internal control, the GAPDH gene(Thermofisher, Hs02786624_g1)

The reverse transcription reaction was performed using the reverse primer and reverse transcriptase (TaqMan-compatible, ThermoFisher N8080234). Real-time PCR was performed in duplex for the GAPDH and Col-I primers and probe sets using TaqMan MasterMix (ThermoFisher 4370048). The cycle threshold or C_*t*_ value, which correlates inversely with the concentration of target mRNA, was determined as the cycle number at which the fluorescence emission of the reporter probe increases above a threshold level. The results from the thermocycler (Applied Biosystems 7300) are reported as the mean GAPDH normalised C_*t*_ for Col-I expression ± standard deviations of three independent experiment performed in triplicate.

### 2.13 Human collagenase MMP-1 ELISA

The collected cell culture supernatants from the PCR experiment were diluted 1:200 in media. The media was used to perform the Human MMP1 ELISA kit (Abcam, ab215083) according to manufacturer's instructions for cell culture supernatants. Results are reported as means ± standard deviations of three independent experiment performed in triplicate.

### 2.14 Swelling measurements

Crosslinked collagen films were weighed then incubated in 5 mL deionised water for 24 h at 37 °C. The hydrated samples were blotted on filter paper, and the hydrated films were subsequently weighed. The water uptake of the material was calculated according to the formula:

$$\text{Water Uptake }\left(\text{\%}\right)=\left(1-\frac{\text{Hydrated mass of sample}}{\text{Original dry mass of sample}}\right)\times 100\text{\%}$$

### 2.15 Optical microscopy

The 24 well plates with cells seeded for 7 days and lysed for PCR were imaged using phase contrast microscopy to identify morphological changes or the presence of shrinkage in the films following cell culture. Images were taken using a Zeiss Axio Observer Z1 microscope with an AxioCam 503 camera (10× objective lens; Carl Zeiss Ltd, Cambridge, UK).

### 2.16 Statistical analysis

Unless otherwise stated α=0.05 for all statistical analyses used. All datasets were evaluated for normality and homoscedasticity using the Anderson-Darling Normality and Levene's tests respectively. Where datasets are noted to be normally distributed and homoscedastic, a one-way ANOVA test was performed followed by a Tukey post-hoc test. Where datasets do not pass the normality and homoscedasticity tests, the Kruskall-Wallis non-parametric test and Mann-Whitney U post-hoc test were employed. * indicates p<α. All statistical annotation indicates the statistical difference between the data point annotated and the non-crosslinked (NonXL) values. Individual *p*-values of each comparison can be obtained from the supplementary files in the Apollo data repository containing raw data.

## 3 Results

### 3.1 Chemical and mechanical characterisation

**Fig. 1:**
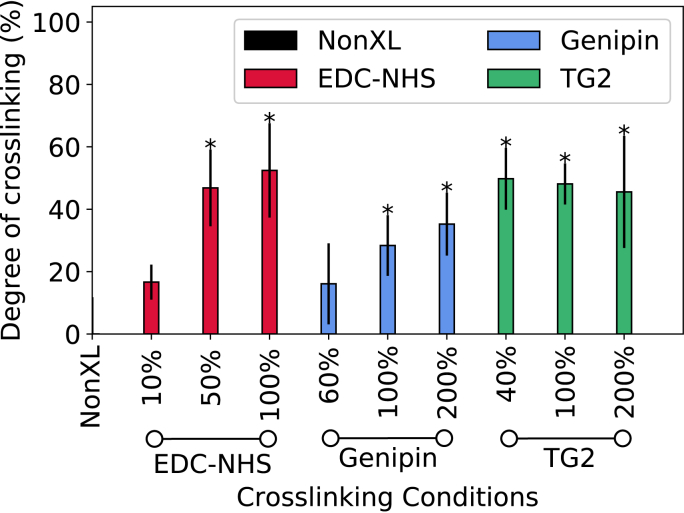
Chemical characterisation of treated collagen films: The ninhydrin assay was used to quantify the degree of collagen film crosslinking using EDC-NHS, genipin and TG2. The percentages on the x-axis represent the concentrations of crosslinking solutions, with the resulting degree of crosslinking (y-axis) depending on the nature of the crosslinking reaction applied. Samples exhibiting a significant difference from the non-crosslinked sample are noted with a *.

The crosslinking conditions are represented with respect to a standard crosslinker solution concentration: the ‘100%’ standards for EDC-NHS, genipin and TG2 are defined in the Materials and Methods (Section 2). The results of the ninhydrin free amine assay, shown in Fig. 1 reveal that with increasing crosslinker concentrations of EDC-NHS and genipin, a greater degree of crosslinking was achieved. At the lowest concentrations of genipin and EDC-NHS, approximately 20% amine crosslinking was achieved. The highest concentrations of EDC-NHS and genipin produced up to 52% and 35% of crosslinking respectively. However, TG2 produced the same degree of amine reduction for all enzyme concentrations tested (~50%). Representative FTIR spectra and triple helical contents measured for the non-crosslinked, EDC-NHS, genipin- and TG2 treated collagen films revealed no statistically significant differences in the triple helical content of the protein, indicating that minimal changes were imparted to the conformation of the protein upon crosslinking (Supplementary Information).

**Fig. 2:**
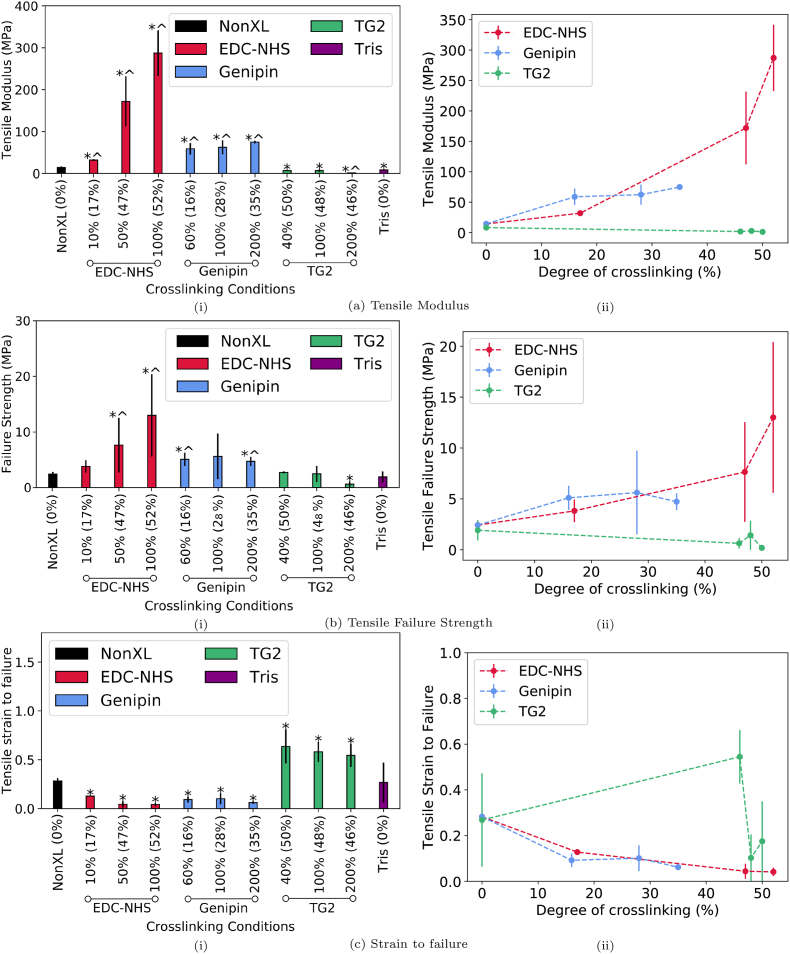
Mechanical characterisation of collagen films crosslinked with EDC-NHS, genipin and TG2: a) Tensile modulus b) Strain at failure and c) Tensile failure. Subfigures (i) represent the data with respect to the concentrations of various crosslinkers. Samples exhibiting a significant difference from the non-crosslinked sample are noted with a *, and from the Tris buffered sample are marked with a ^. Values outside brackets represent crosslinker concentration whereas values in brackets represent the ‘degree of crosslinking’ as determined by the ninhydrin free amine assay for a given condition. Subfigures (ii) summarise the data as a function of the ’degree of crosslinking’.

The tensile modulus, strain to failure and ultimate tensile strength of the collagen films were also measured to characterise the impact of chemical crosslinking on the mechanical properties of the films (Fig. 2a, b and 2c). Representative stress-strain curves are provided in the Supplementary Information. The tensile test data revealed that there was a significant increase in the tensile modulus upon crosslinking with all conditions of EDC-NHS and genipin by up to 2000% and 400% respectively at the highest concentrations. For the same degree of crosslinking of approximately 20%, genipin offers a slightly higher stiffness in comparison to its EDC-NHS counterpart. On the other hand, the tensile modulus decreased by up to 80% upon crosslinking with TG2. Furthermore, EDC-NHS and genipin-treated films withstood significantly higher tensile stresses (by up to 400% and 130% respectively) when compared with the non-crosslinked samples. TG2 treated films demonstrated no significant improvement in strength at low crosslinking concentrations, and a significant 66% reduction in the failure strength at the highest crosslinking concentrations (200%). With the notable exception of TG2 treatment where up to a 140% increase was observed in the strain to failure, all other conditions of treatment resulted in a reduction of the strain at failure by up to 85%.

**Fig. 3:**
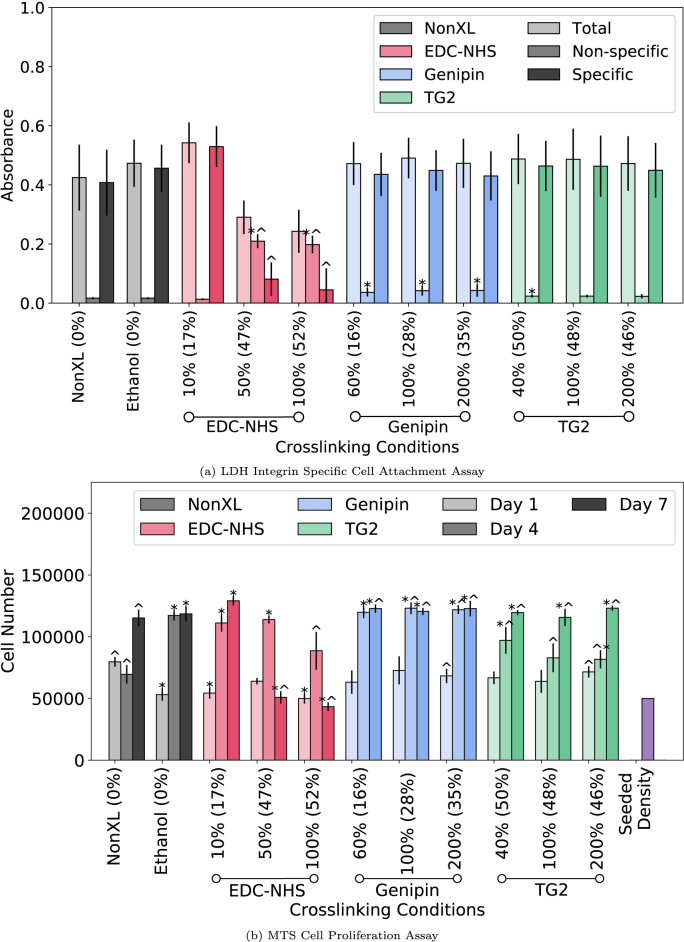
Integrin mediated adhesion of human dermal fibroblasts and cellular proliferation over 7 days in culture. (a) LDH attachment assay performed on non-crosslinked and EDC-NHS, genipin and TG2 crosslinked films. Integrin-specific and non-specific binding were separated through the addition of EDTA or MgCl^2^ to cell media. (b) Cell proliferation over 7 days was evaluated using the MTS assay. A * indicates p < 0.05 with respect to the non-crosslinked condition whereas ^ indicates p < 0.05 with respect to the ethanol treated conditions. Values outside brackets represent crosslinker concentration whereas values in brackets represent the ‘degree of crosslinking’ as determined by the ninhydrin free amine assay for a given condition.

The effect of incubating films in Tris buffer at 37 °C for 24 h on the mechanical properties was also evaluated as a control. A reduction in modulus from the non-crosslinked film was noted for all concentrations of TG2 crosslinking and Tris buffered films. Only the highest concentration of TG2 exhibited a significant reduction from the Tris buffered samples. The TG2 crosslinked films for tensile testing also exhibited significant shrinkage post crosslinking. This shrinkage was observed when the collagen films were placed in pure Tris buffer at 37 °C but not in Type II de-ionised water at 37 °C.

### 3.2 Cell attachment and proliferation

Cellular adhesion via integrin receptors present on the surface of human dermal fibroblasts to the GXOGER motif present on collagen fibres was investigated. Here, fibroblasts were added to non-crosslinked and EDC-NHS, genipin and TG2 treated collagen films in the presence of either Mg^2+^ or EDTA. Values for total adhesion (Mg^2+^-dependent), non-specific (EDTA) and specific (Mg^2+^−EDTA) are displayed in Fig. 3a.

Data from EDC-NHS treated films revealed a statistically significant increase in non-specific binding with increasing crosslinker concentrations (50% and 100%) and a decrease in integrin specific binding. All genipin crosslinked films, and 40% TG2 crosslinked films have a small but statistically significant increase in non-specific binding in comparison with non-crosslinked and ethanol crosslinked films. The overall, integrin specific cell attachment remains largely unaffected across all concentrations of genipin and TG2 crosslinked films when compared with non-crosslinked control samples, consistent with an intact GXOGER motif on the collagen fibres with these two crosslinkers.

The effects of crosslinking collagen using EDC-NHS, genipin and TG2 on HDF proliferation were assessed using the MTS assay (Fig. 3b). Over the seven-day period, no significant proliferation was observed with the non-crosslinked collagen films. An increase was noted with the Day 4 proliferation values of EDC-NHS crosslinked and genipin crosslinked films, as well as the ethanol control when compared with untreated collagen films. The Day 4 proliferation values on TG2 films and 100% EDC-NHS crosslinked films were not significantly different from the non-crosslinked condition. EDC-NHS films revealed a sharp decrease in the proliferation on Day 7, to a value significantly lower than the cell numbers observed for non-crosslinked films. A similar decrease was also seen with the ethanol controls at Day 7. Although there was no significant increase in the total or specific cell attachment with the TG2 treated samples, by day 7 a significant increase in proliferation was also observed when compared with the non-crosslinked samples. Both the untreated and treated films cast on the well-plates were stable over the seven-day cell culture period and displayed negligible swelling or shrinkage (Supplementary Information, Figs. 8 and 10).

The gene expression level of the ECM protein Collagen-I, by the seeded human dermal fibroblasts was found to be unaffected by the crosslinking of the substrates (Supplementary Information). The expression levels of matrix metalloproteinase-1 (MMP-1), a collagenase that can degrade ECM proteins was noted to increase with increasing concentrations of EDC-NHS and TG2-treatment (Supplementary Information). Interestingly, this was not observed for genipin crosslinking where MMP-1 levels were found to decrease with increasing crosslinking concentration.

### 3.3 Cytotoxicity

**Fig. 4:**
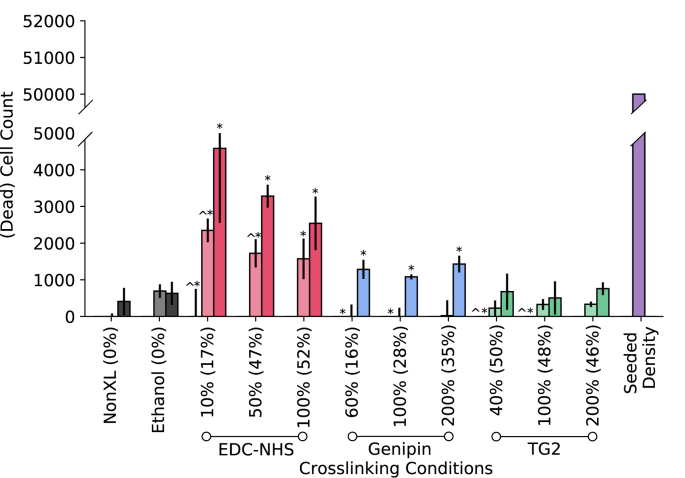
Cytotoxicity analysis of human dermal fibroblasts cultured on chemically crosslinked collagen films. (a) The supernatant of cells cultured on the crosslinked films over a 7 day period was analysed using the LDH metabolic activity assay. A * indicates p < 0.05 with respect to the non-crosslinked condition whereas ^ indicates p < 0.05 with respect to the ethanol treated conditions. Values outside brackets represent crosslinker concentration whereas values in brackets represent the ‘degree of crosslinking’ as determined by the ninhydrin free amine assay for a given condition.

To evaluate whether the decrease in cell number corresponded to cell death from encountering a cytotoxic environment, an LDH assay was performed on the supernatant medium from the MTS cell proliferation assay. While some metabolic activity was noted with ethanol treatment and crosslinking at Day 4 and 7, as seen in Fig. 4, cell death was negligible for all conditions given the initial number of seeded cells.

**Fig. 5:**
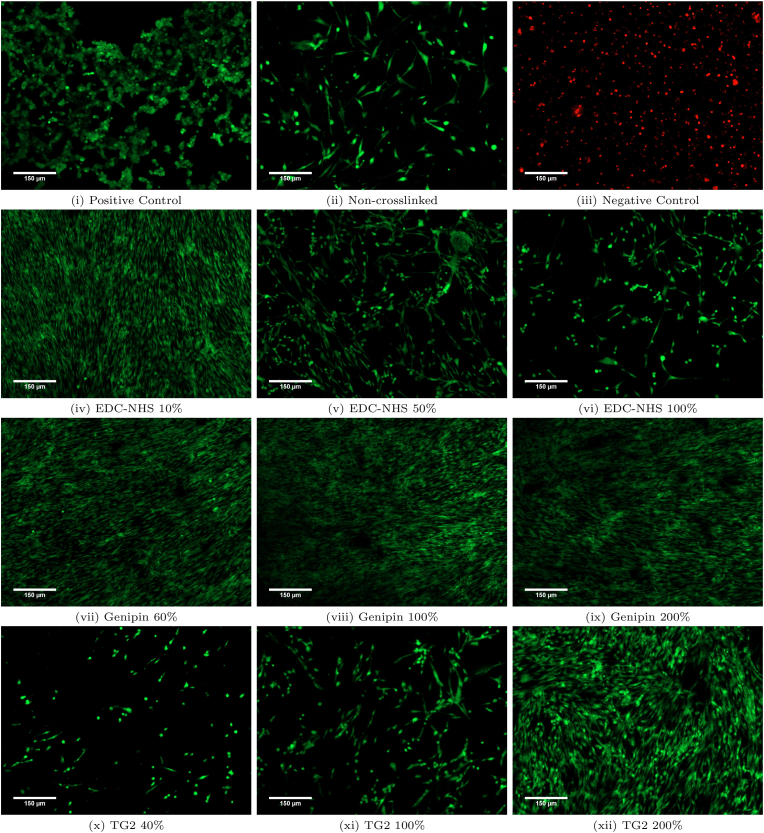
Cell viability on the non-treated and crosslinked films assessed using the LIVE/DEAD assay. Fibroblasts grown on tissue culture plastic was used as a positive control and fibroblasts treated for 20 min with 3% glutaraldehyde was used as a negative control. Red: EthD, green: Calcein. (For interpretation of the references to colour in this figure legend, the reader is referred to the Web version of this article.)

These observations were further supported by the use of the live/dead assay. Fig. 5 shows fluorescence microscopy images from Day 7 of the assay, where live cells appear green due to the retention of esterase in intact cell membranes (positive control) and dead cells appear red due to the infiltration of ethidium bromide-1 and staining of the DNA though their damaged cell membranes (negative control) during apoptosis. Little to no apoptotic cells were observed on any of the crosslinked films over the course of 7 days, and nearly all cells were viable on the differently treated films. Most notably, HDFs were able to grow homogeneously on the 10% EDC-NHS crosslinked samples. Films crosslinked with higher concentrations of EDC-NHS also contained all live cells (in green), but were more sparsely colonised. For the genipin treated films cells grow homogeneously and blanket the entire film at all crosslinking conditions with live cells. For the TG2 treated films, less live cell coverage of the films was observed at the lowest crosslinking conditions, with homogeneous coverage of cells observed at the higher crosslinking conditions. Overall, this assay confirmed that very low levels of cell death was observed for any cells adhered onto the surface of all films over a seven-day period (Supplementary Information, Figs. 4 and 5 for Day 1 and 4 images).

### 3.4 Cell spreading

**Fig. 6:**
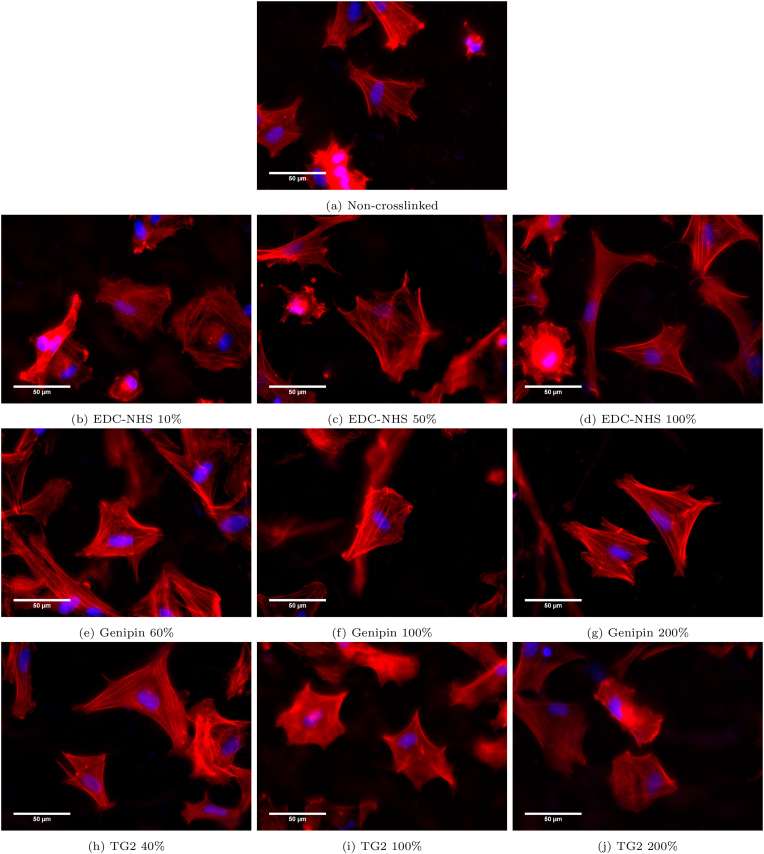
Effect of crosslinking treatments on human dermal fibroblast morphology and spreading. Fibroblast morphology is imaged on non-crosslinked and EDC-NHS, genipin- and TG2-treated collagen films. HDFs are observed to cluster and demonstrate a rounded morphology with increasing EDC-NHS crosslinking, expressing a spindle-like morphology of the filopodia (labelled in Fig. 7a) on the 50% and 100% EDC-NHS treated films. HDFs present in all other conditions are well-spread exhibiting broad lamellipodia. Fibroblasts on genipin and TG2-treated films also possess prominent ventral stress fibres (labelled in Fig. 7b). Genipin-treated films autofluorescence of collagen fibres in the red channel. Red: Phalloidin (actin), blue: DAPI (nucleus). (For interpretation of the references to colour in this figure legend, the reader is referred to the Web version of this article.)

A cell spreading study was conducted to characterise the morphology of HDFs on non-crosslinked as well as EDC-NHS, genipin and TG2 treated films as seen in Fig. 6.

**Fig. 7:**
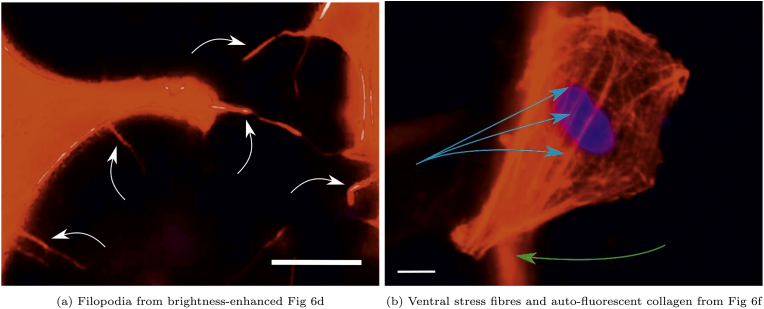
Labelled morphological features observed in attached human dermal fibroblasts in Fig. 6 a) filopodia (white arrows) extending from the cells to the substrate in EDC-NHS crosslinked samples b) ventral stress fibres (blue arrows) visible on the genipin and TG2 crosslinked films. White scale bar on images corresponds to 10 m. Fibres of genipin crosslinked collagen also auto-fluoresce (green arrow) in the phalloidin channel. (For interpretation of the references to colour in this figure legend, the reader is referred to the Web version of this article.)

Several qualitative differences in the cell morphology can be observed between the treatments. For the EDC-NHS treated films, some single cells were well-spread on the film surfaces, whereas others have begun their apoptotic detachment from the sample surface. (Fig. 6c and d). A greater incidence of such apoptotic cells lifting off in EDC-NHS treated films was observed at the higher concentrations of EDC-NHS. The spindle-like morphology of the filopodia as labelled in Fig. 7a were observed in 50% and 100% EDC-NHS treated films. Such filopodia were not noted in any other condition barring the lowest concentration of TG2 (Fig. 6h).

For all other conditions of crosslinking, broad lamellipodia was observed on the cells, with some tethers extending from the periphery. Genipin crosslinked collagen fibres exhibited autofluorescence in the red [31] channel, allowing the collagen fibres on to which the cells have adhered to also be observed. These strands of auto-fluorescing collagen fibres labelled in Fig. 7b were observed in Fig. 6e, f, 6g. Though very few dorsal stress fibres are observed on the fibroblasts, ventral actin stress fibres are prominent in all HDF adhered to genipin and TG2 treated films, as labelled in Fig. 7b.

The data obtained here were also quantified in terms of the area, perimeter, aspect ratio, circularity and roundness of a single cell (Details provided in Supplementary Information, Table 1).

## 4 Discussion

Among the cues responsible for guiding cell attachment, surface biochemistry and mechanical properties represent two of the most significant stimuli presented by the substrate. In this study, we attempt to separate the effects of the ligands available and the mechanical properties of the collagen based constructs by characterising the chemical and mechanical properties of the crosslinked films and evaluating short-term and long-term effects of the treatments on human dermal fibroblast attachment and proliferation.

### 4.1 Chemical and mechanical characterisation

The EDC-NHS, genipin and TG2-treated films were characterised using the ninhydrin assay to characterise the changes to the free amines in the underlying collagen structure. By colorimetrically analysing the purple ‘Ruhemann's dye’ released in the process [30], the ninhydrin free amine assays revealed that all treatments resulted a reduction in the free amines in collagen.

Previous work on 100% EDC-NHS collagen films revealed that tensile modulus increased nearly six fold upon crosslinking [32] when measured using both a tensile and compression testing rig [33] for films and scaffolds respectively. Although many mechanical properties have been characterised including storage and loss moduli for collagen gels crosslinked by genipin [23,34] or shear resistance for TG2 crosslinked fibrils [35], there has been little work on the tensile modulus for TG2-and genipin-treated insoluble collagen films. Here we note a five-fold increase in the tensile modulus after crosslinking with EDC-NHS at the lower end, and an improvement of nearly two orders of magnitude at the higher end of the EDC-NHS treated films and over an order of magnitude increase in modulus with genipin treated films (Fig. 2a). Similarly, this work builds on previous comparisons of genipin and EDC-NHS crosslinking of chitosan-collagen-elastin films [36] by normalising the mechanical properties obtained as a function of the free amines reduction or the ‘degree of crosslinking’. The tensile modulus for genipin-treated samples is slightly higher than the EDC-NHS equivalent at a given degree of crosslinking. However, the mechanical stiffness range covered by genipin lies in the middle of the crosslinking range offered by EDC-NHS. This presents genipin as a viable alternative for EDC-NHS where similar mechanical properties are desired. Additionally, in this work, the thickness of each tested film was measured using optical microscopy, enabling more accurate measurements of mechanical properties to be made as compared with previous works which use an average thickness for a given condition [32].

As first explored in the introduction, a reduction in free amines may occur either through the formation of amide crosslinks or the amine reduction may also occur via a conversion of glutamines to glutamates for the TG2 treated samples. Although TG2 has been noted to increase the modulus in freeze-dried neutralised soluble collagen samples [26], this work demonstrates for the first time, the global mechanical properties of insoluble collagen films that have been treated with TG2. Here, the modulus decreased at all concentrations of TG2 when compared with the non-crosslinked condition. A characterisation of these films using atomic force microscopy of TG2-treated films demonstrated that locally, TG2 improved the mechanical properties of collagen at low concentrations but not at 200% [37]. This is consistent with the decreased elastic modulus at very high TG2 concentrations observed in this paper. The macroscopic mechanical behaviour observed here is likely to arise predominantly from the effect of the Tris buffer, since a similar reduction in modulus is also noted for the Tris buffered samples. Collagen hydrolysis occurs at temperatures as low as 5 °C at pH values above 4.5 over a 24 h period [38]. Some reduction in the modulus may therefore be attributed to hydrolysis of collagen at pH 7.4 for 24 h at 37 °C. However, the Tris buffer may also have a part to play in the pronounced reduction, with evidence in literature suggesting its ‘non-innocent’ role as a buffer via the formation of adducts with reduced collagen [39].

However, further reduction in modulus at higher concentrations of TG2 is unexpected when compared with the soluble collagen counterparts which are used in collagen-based hydrogel networks. By taking into account the reduction in free amines, this behaviour can be resolved through the hydrolytic pathway that can be adopted by TG2 in the presence of few amines [40,41]. From the results obtained here, it seems likely that the relative immobility of collagen in the final cast film results in a conversion of glutamines to glutamates as opposed to the selective amidation of the glutamines (Supplementary Information ), unlike the mobile fragments of collagen in solution. This is further supported by previous evidence with microbial transglutaminase where the extent of crosslinking has been shown to depend on the extent of denaturation of collagen that takes place at high temperatures, allowing for the active sites within collagen to be accessible to the transglutaminase [42]. In this study, the lack of significant denaturation, and the fibrillar nature of the collagen further support the suggestion that collagen in a fibrillar form is an unsuitable substrate for significant crosslinking. As a result, we can conclude that three films of varying characteristics were created: stiff films with reduced glutamates (EDC-NHS), compliant films with some increase in glutamates (TG2) and stiff films with no reduction in glutamates (genipin).

### 4.2 Integrin-specific attachment and fibroblast proliferation

From the HDF attachment assay, EDC-NHS crosslinking was noted to significantly ablate integrin-specific motifs in collagen, resulting in a reduced specific binding at high concentrations of EDC-NHS. This result is consistent with other observations of integrin specificity with the integrin I domain adhesion [21] and anchorage dependent cells such as HT1080 human fibrosarcomas, human platelets [43] and rat glioma (Rugli) cells [21,28].

Previous work on the integrin-mediating binding to genipin-crosslinked constructs have suggested a decrease in integrin-binding since genipin may crosslink to arginines, resulting in the loss of RGD motifs [44]. In this work, although genipin resulted in some significant non-specific binding, the scale of this effect is much lower than its specific binding, and the non-specific binding of EDC-NHS treated films. The differences between the behaviours can be reconciled by the cryptic nature of RGD motifs in triple helical collagen-I, where GXOGER serves as the primary MIDAS motif for integrin-mediated binding [45]. Thus, the integrin-specific binding through GXOGER motifs can be concluded to be unaffected by genipin crosslinking in this work.

TG2-treatment of soluble collagen-I also did not result in any changes to integrin specific binding. Therefore, although it was hypothesised that an increase in glutamates may increase integrin specific binding, this result suggests that the number of glutamates created are either insignificant in their total number, or do not present a significant contribution to the initial short-term attachment of fibroblasts. In the TG2-crosslinked collagen constructs evaluated in the literature, β integrin expression has been reported to be increased after treatment [17]. In these scaffolds however, the result is expected to arise from the presence of TG2 in the scaffolds, in addition to the changes imparted by TG2 to the collagen structure [17].

In addition to the glutamic acid residue of the MIDAS motifs, the accessibility of the integrins has also been shown to be affected by steric hindrance surrounding the integrin motifs in triple helix-mimicking proteins [46]. While any crosslinks formed by TG2 and EDC-NHS are ‘zero-length’, genipin is incorporated into the collagen crosslinks. As seen in Fig. 3a, though the inclusion of a large molecule such as genipin does not appear to affect the accessibility of the integrin sites, and the treatment with TG2 also leaves the integrin sites unaffected as compared with the non-crosslinked Collagen-I control.

The long-term effects of the availability of ligands and the mechanical properties of a film were further investigated through the MTS cell proliferation assay (Fig. 3b). The comparison of the behaviour of ethanol treated and EDC-NHS crosslinked samples suggests that there is a delayed effect of the ethanol on the collagen films and consequently the cells. The additional impact of reduced specific binding may also explain the further reduction in proliferating cells at 100% EDC-NHS at Day 4 and 7. Ethanol has previously been used to create esterification in collagen [47]. The esterification was noted to improve cell activity and survival of the pancreatic cells tested, much akin to the short term response observed from Day 1–4 with the ethanol controls. Since esterification requires the use of the carboxylic acids (E and D) in the collagen, the lowered proliferation effect can be associated with the lack of availability of suitable ligands for integrin specific binding over the seven-day period.

This behaviour is also consistent with the increase in proliferation seen with TG2 over the untreated samples, due to the increased glutamates available upon TG2 treatment. As a result, the long-term proliferation can be concluded to be dependent primarily on the availability of appropriate ligands, even if short-term proliferation may be affected by mechanical stiffness as evidenced by the high rates of proliferation from Day 1–4 for genipin and EDC-NHS crosslinked samples.

Although this study characterised the proliferation of HDFs on crosslinked surfaces, there is further scope to investigate whether the observed behaviour arises from a direct interaction with the crosslinked collagen, or an indirect interaction through the ECM deposited on the treated surface. It is hypothesised that at Day 1, cells interact directly with the crosslinked films, but begin to deposit their own extracellular matrix by Day 4. In normal human wound healing, the cell attachment protein fibronectin can be detected histologically at Day 4, with collagen-I and collagen-IV detected at Day 7 [48].

In this work, the data from Day 7 revealed no difference in the gene expression levels of collagen I production by fibroblasts on the crosslinked films (Supplementary Fig. 6), indicating that fibroblast function was normal. However, an increase in the levels of MMP-1, a protease is involved in the degradation of collagen was detected for the EDC-NHS and TG2-treated substrates (Supplementary Fig. 7a). Increase in MMP-1 production has been found to be well-correlated with the effect of strain experienced by the human dermal fibroblasts [49] and the stiffness of the substrates [50]. MMP-1 levels can also be affected by the type of ECM protein [51], and the type of substrate material, independently from the substrate stiffness [52]. As a result, the increase in MMP-1 levels observed here may arise from the deviation from an ideal matrix stiffness, differences in the deposition of other ECM proteins, or changes to underlying chemical structure of collagen. Interestingly, the increase in MMP-1 levels with TG2-treatment observed in this work is also heavily contrasted with the decrease in MMP-1 levels observed with TG2-crosslinked soluble collagen scaffolds in the literature [26], which may arise either due to the lack of residual TG2 in our collagen films, or due to the differences in chemical crosslinking by TG2 in soluble and insoluble collagen. An in-depth investigation of ECM deposition and remodelling is outside the aims and direct scope of this study and provides a further avenue of exploration.

### 4.3 Cytotoxicity of crosslinking treatments

The cytotoxicity of the treatments was evaluated using the live/dead stain and LDH assay on the supernatants of the MTS proliferation assay. As seen from the representative images in Fig. 5, the viability of fibroblasts is not affected by the ethanol, EDC-NHS, genipin or TG2 treatments. As seen in Fig. 4, there were few non-adherent cells on the surface of all treated films. The extent of cell death across the seven-day period also mirrors the trends seen with the proliferation seen in Fig. 3b. However, the cell numbers in the LDH assay were over an order of magnitude lower than the total seeded density on all films. This suggests that though the treatments can affect cell death of non-adherent cells, the overall cytotoxicity of the treatments is negligible.

The increase in cell proliferation, with little apoptosis observed with the stiffened treated films has been previously noted with fibroblasts in crosslinked substrates of tunable mechanical properties [53,54]. Although the cellular attachment and proliferation results presented here pertain to thin insoluble collagen films cast which are immobilised on well plates and therefore are expected to show limited swelling (Supplementary Information, Figs. 8 and 10) and cell infiltration, a comparison of 2D and 3D behaviour on gelatin constructs revealed that similar trends were observed in two dimensional hydrogel films but not in three dimensional hydrogel matrices [55].

### 4.4 Fibroblast spreading and morphology

The lack of variation in the overall cell spreading behaviour, excepting the greater incidences of rounded cells and cellular projections at higher crosslinking concentrations of EDC-NHS, indicates that the contact behaviour of the cells are largely dependent on the availability of ligands on a substrate. This is akin to the behaviour seen when human gingivial fibroblasts are seeded on fibrillar collagen with localised integrins [56]. The presence of filopodia has also been suggested to be driven by the matrix compliance where the ability to form larger focal adhesions and lamellipodia is reduced [57]. Therefore, the extension of long and thin filopodia at the highest levels of EDC-NHS crosslinking is consistent with the simultaneous decrease in available integrin specific cell binding sites (Fig. 3a), and an increase in substrate rigidity (Fig. 2a).

### 4.5 Proposed mechanism of action

**Fig. 8:**
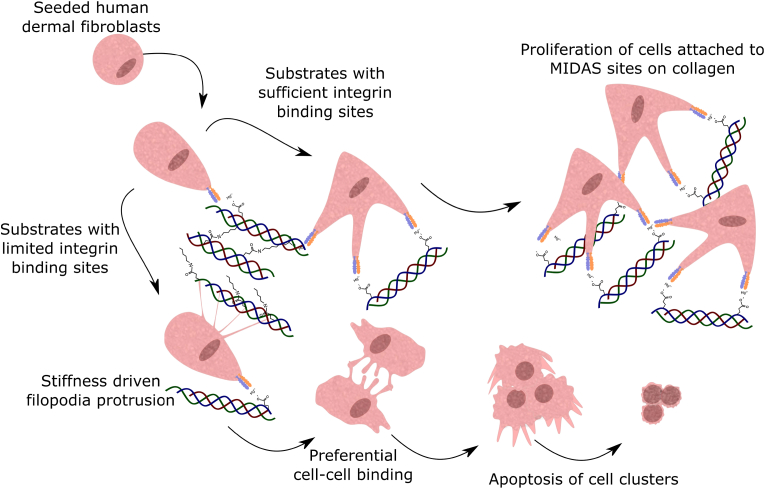
Proposed mechanism for human dermal fibroblast attachment and proliferation on collagen films. Initial attachment and spreading is predominantly driven by adhesion to integrin specific binding sites on the collagen film. Integrin-adherent cells then proceed to proliferate, with the rate driven by the stiffness of the underlying substrate. Where such motifs are engaged in crosslinking and unable to sustain the divalent metal cation needed for adhesion via the integrins, filopodia are extended to probe for substrate rigidity. Cells that have adhered to the surface in this manner, preferentially adhere to other cells. These cell clusters exert sufficient force to detach from the film surface, thus eventually undergoing apoptosis and lifting off the substrate surface.

As a result, we propose the mechanism shown in Fig. 8 for human dermal fibroblast attachment and proliferation. Human dermal fibroblasts begin spreading along the collagen surface, forming cell projections with specific binding sites on the film. In collagen substrates where there is insufficient specific binding sites available for further cell attachment, such as the EDC-NHS treated films, cell-cell junctions are preferentially formed. Cell clusters formed on these surfaces begin their apoptotic cycle, until they lift off the surface of the film, into the supernatant as seen in Fig. 4. Conversely, fibroblasts strongly anchored onto the collagen surface begin proliferating in response to the stiffness of the substrate.

## 5 Conclusions

In this body of work, collagen constructs were crosslinked using EDC-NHS, genipin and tissue transglutaminase to explore three elements of the collagen crosslinking process. EDC-NHS results in an extremely high tensile modulus, at the expense of integrin-specific attachment and long-term cell proliferation through the loss of glutamates in the crosslinking process.

At the same degree of crosslinking, genipin-treated films offer remarkably high cell activity, and a comparable tensile modulus with EDC-NHS treated films. Following this work, genipin therefore stands out in its potential to replace EDC-NHS where medium to high mechanical stiffness and excellent cell attachment and proliferation are desired. Tissue transglutaminase may promote the conversion of pre-existing crosslinks in insoluble collagen to glutamines at high concentrations, resulting in low stiffness substrates that maintain higher cell attachment and proliferation than untreated samples. The use of three different crosslinking chemistries bring to light the relative importance of mechanical and biochemical cues on human dermal fibroblasts. Although short-term proliferation may be driven by mechanical cues, long-term stable proliferation of HDFs are correlated with high levels of integrin-specific binding, irrespective of the modulus post-treatment.

The crosslinking process therefore represents a pathway for access to different regimes within the property space of tensile modulus as well as cell attachment and proliferation. As a result, the potential to create structures of tunable mechanics and bioactivity in collagen can be exclusively facilitated through the choice of crosslinker chemistry, further expanding the versatility of collagen within tissue-engineering scaffolds.

## CRediT authorship contribution statement

**Malavika Nair:** Methodology, Investigation, Formal analysis, Writing - original draft, Visualization. **Ramneek K. Johal:** Methodology, Conceptualization, Investigation, Supervision, Writing - review & editing. **Samir W. Hamaia:** Investigation, Writing - review & editing. **Serena M. Best:** Conceptualization, Writing - review & editing, Supervision, Project administration, Funding acquisition. **Ruth E. Cameron:** Conceptualization, Writing - review & editing, Supervision, Project administration, Funding acquisition.

## Declaration of competing interest

The authors declare that they have no known competing financial interests or personal relationships that could have appeared to influence the work reported in this paper.

## Data Availability

Data are available on the University of Cambridge Apollo Open Data Repository at https://doi.org/10.17863/CAM.36098.
