# Supplementary material for: Tunable bioactivity and mechanics of collagen-based tissue engineering constructs: A comparison of EDC-NHS, genipin and TG2 crosslinkers
Source: Biomaterials. 2020 Sep;254:120109. doi: 10.1016/j.biomaterials.2020.120109 (PMC7298615; doi:10.1016/j.biomaterials.2020.120109)
Supplement: Multimedia component 2 [file mmc2.pdf]

Tunable bioactivity and mechanics of collagen-based tissue engineering constructs: A comparison of EDC-NHS, genipin and TG2 crosslinkers.

1. Crosslinking mechanisms

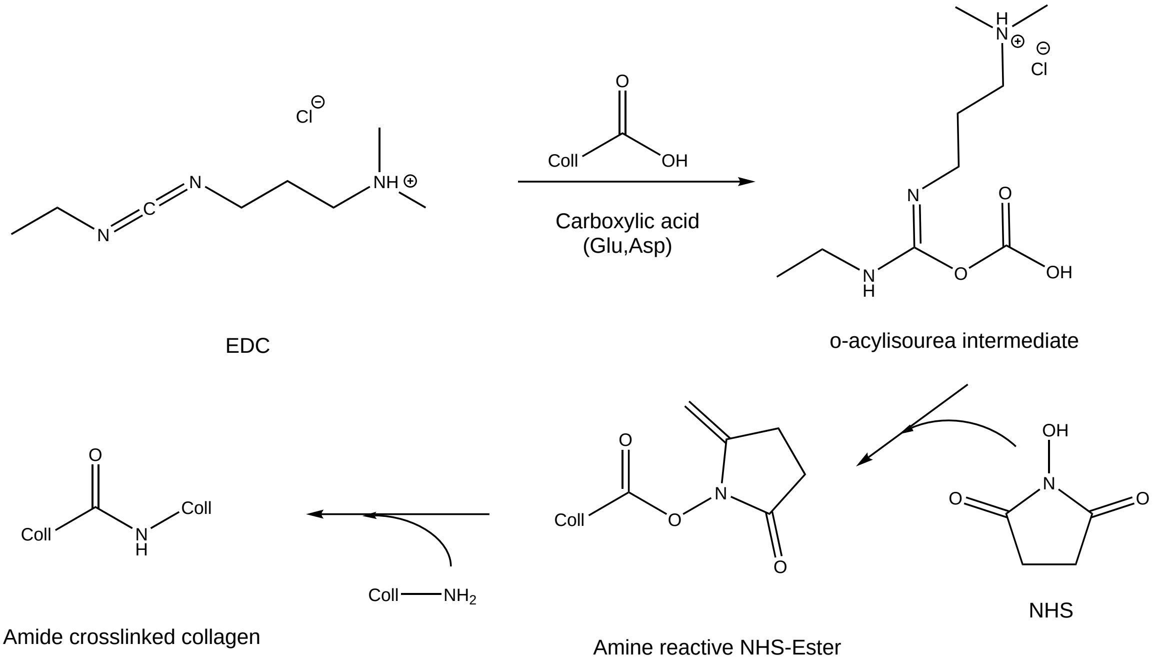

(a) EDC-NHS

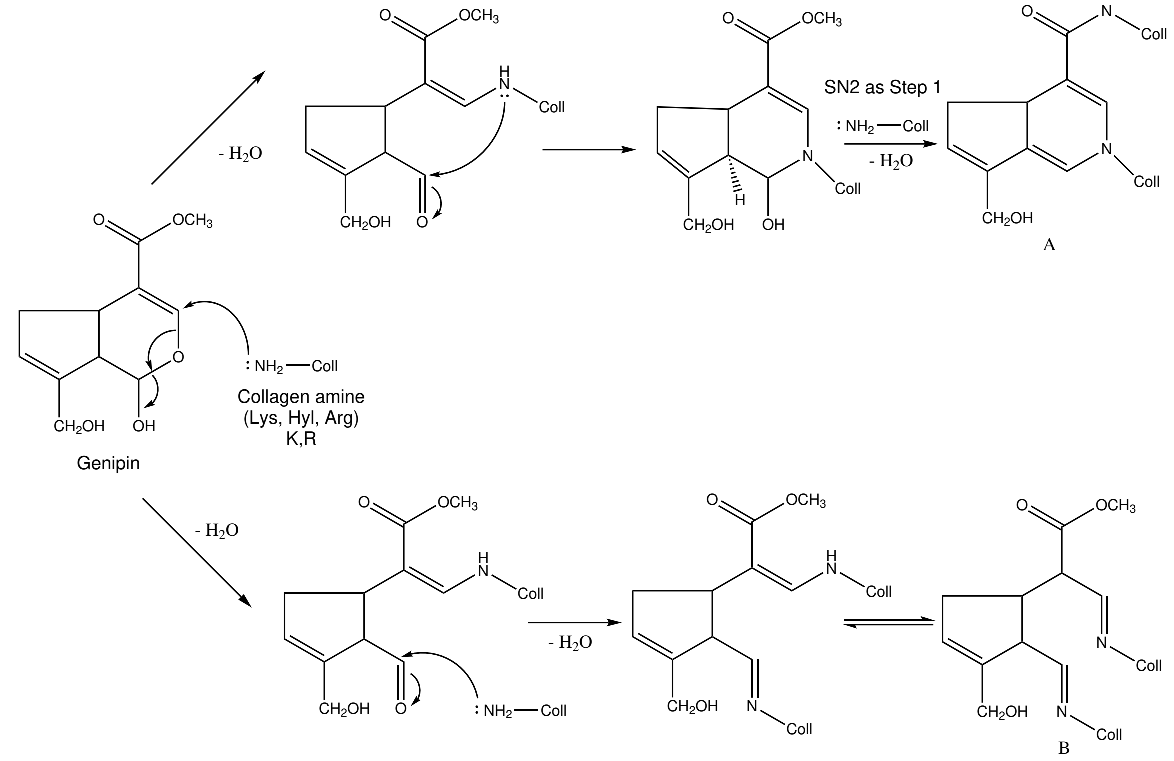

(b) Genipin

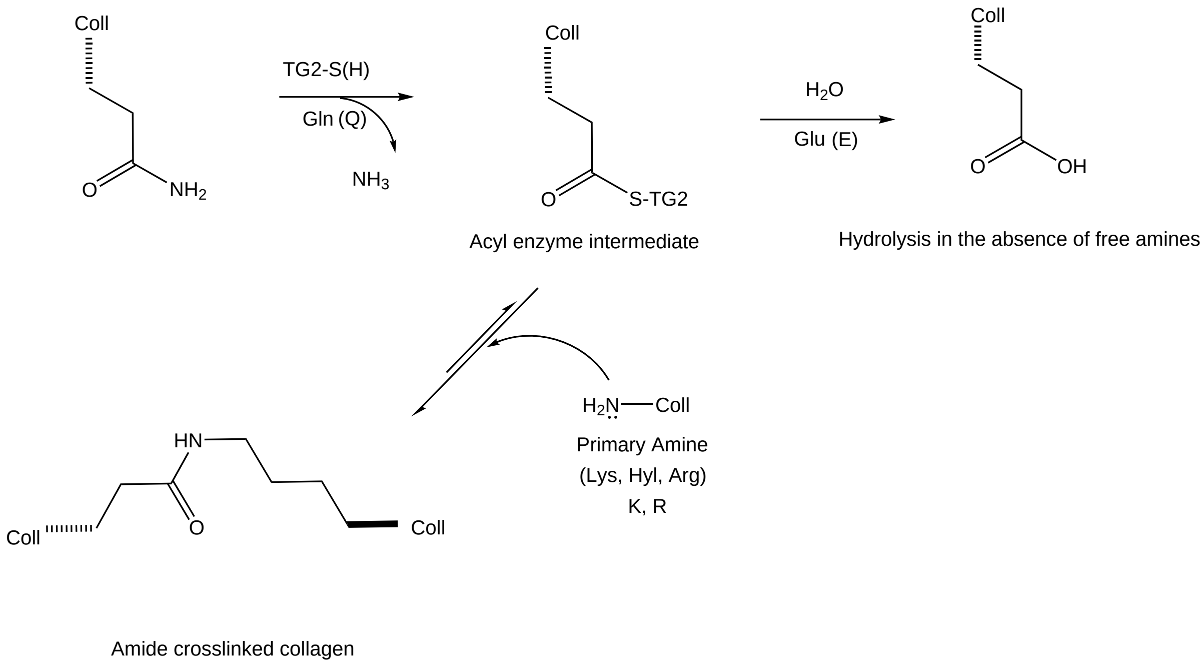

(c) Transglutaminase

Figure 1: Crosslinking mechanisms of EDC-NHS (1a), genipin (1b) and tissue transglutaminase (1c). EDC-NHS crosslinks via an amide mechanism, making use of the glutamates (E) in the amino acid sequence. These glutamates form an integral part of the GFOGER metal ion-dependent adhesion sites in collagen for integrin specific binding. Unlike EDC-NHS, genipin and transglutaminase, bypass the use of aspartates and glutamates and crosslink solely via the primary amines. Where transglutaminase is unable to find suitable amines for crosslinking, glutamines and some existing amides are converted into glutamates (E).

2. Fourier-transform Infrared Spectroscopy

Representative FTIR spectra of the non-crosslinked, EDC-NHS, genipin- and TG2 treated collagen films are shown in Figure 2a. As seen in Figure 2b, no statistically significant differences are observed across the data sets in the triple helical or α-helical components of the protein, indicating little change to the triple helical conformation. The proportion of amide I peak occupied by the triple helical peak is also consistent with values obtained in the literature.[1]

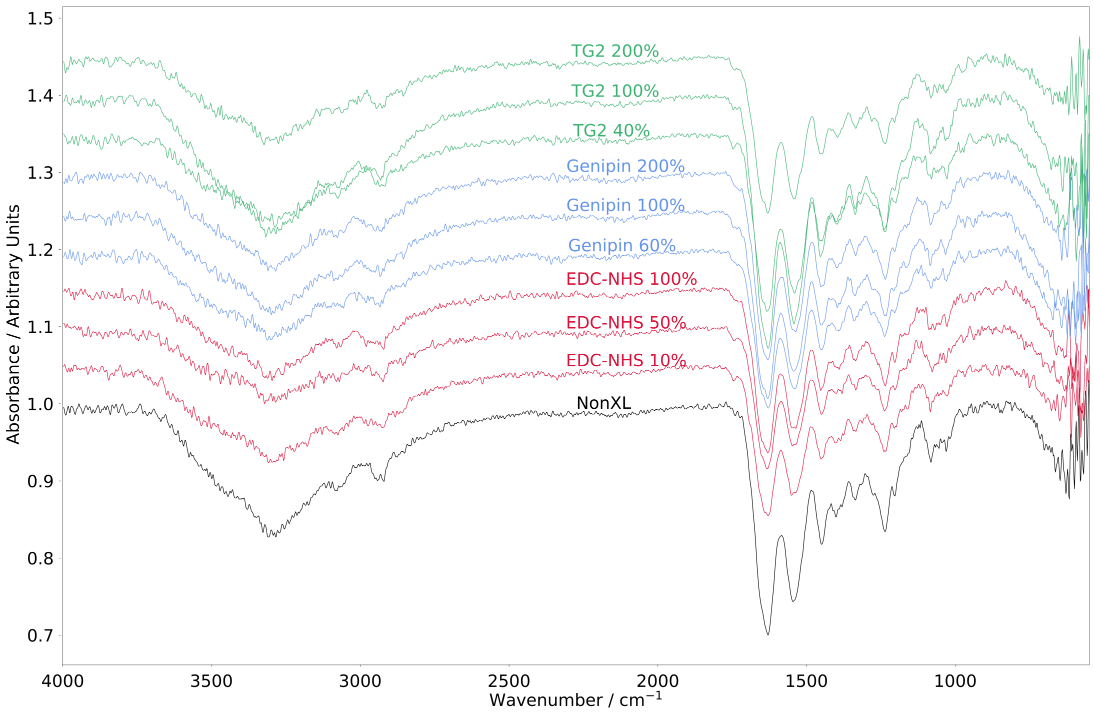

(a) Representative FTIR spectra

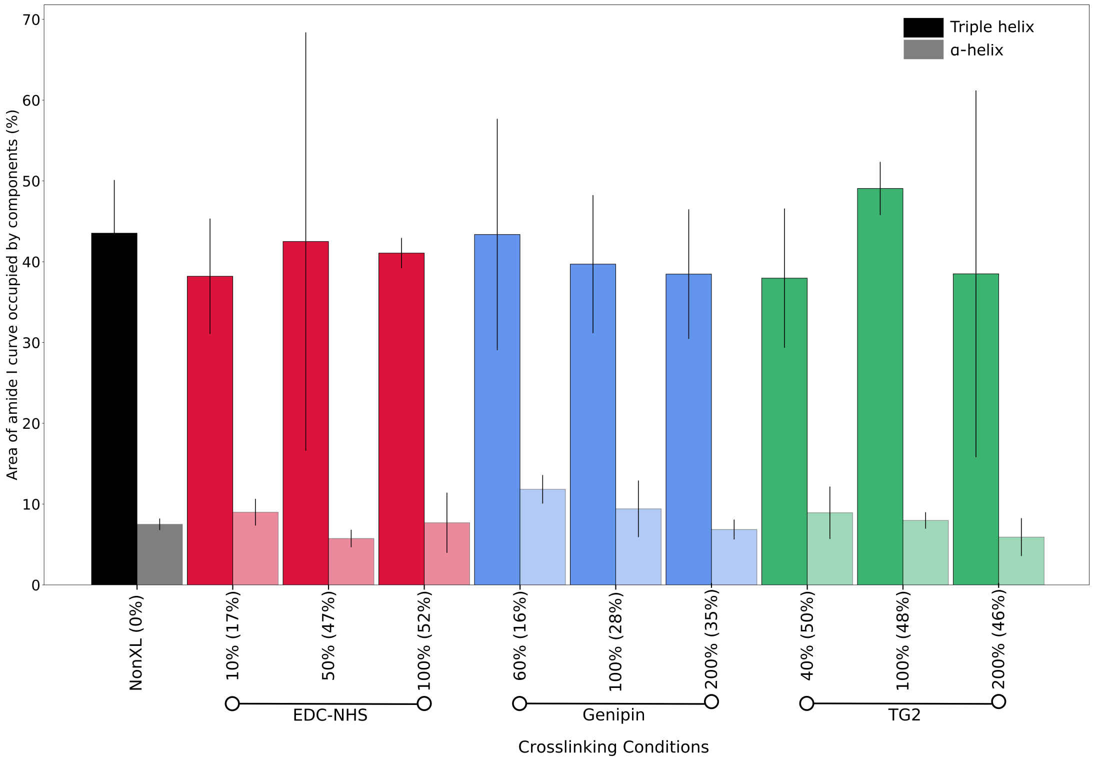

(b) Triple helical content

Figure 2: FTIR spectral data for untreated, EDC-NHS, genipin- and TG2-treated films. a) Representative FTIR spectra for one sample from each crosslinking condition. Spectra are offset in absorbance for clarity. b) Triple helical content (as a proportion of the total amide peak) are not significantly affected by the EDC-NHS, genipin and TG2 treatment.

3. Representative stress-strain curves

Representative stress-strain curves of the collagen films under tension are illustrated in Figure 3. A typical curve presents four regions: an initial toe region, linear-elastic extension, plastic deformation and finally catastrophic failure. The toe region is present in all treated samples, although it is less pronounced in the highly crosslinked films, particularly Genipin at 200% and EDC-NHS 100%. Although all films also demonstrated limited plastic deformation prior to failure, a short plateau region was visible for the non-crosslinked and 60% genipin crosslinked films.

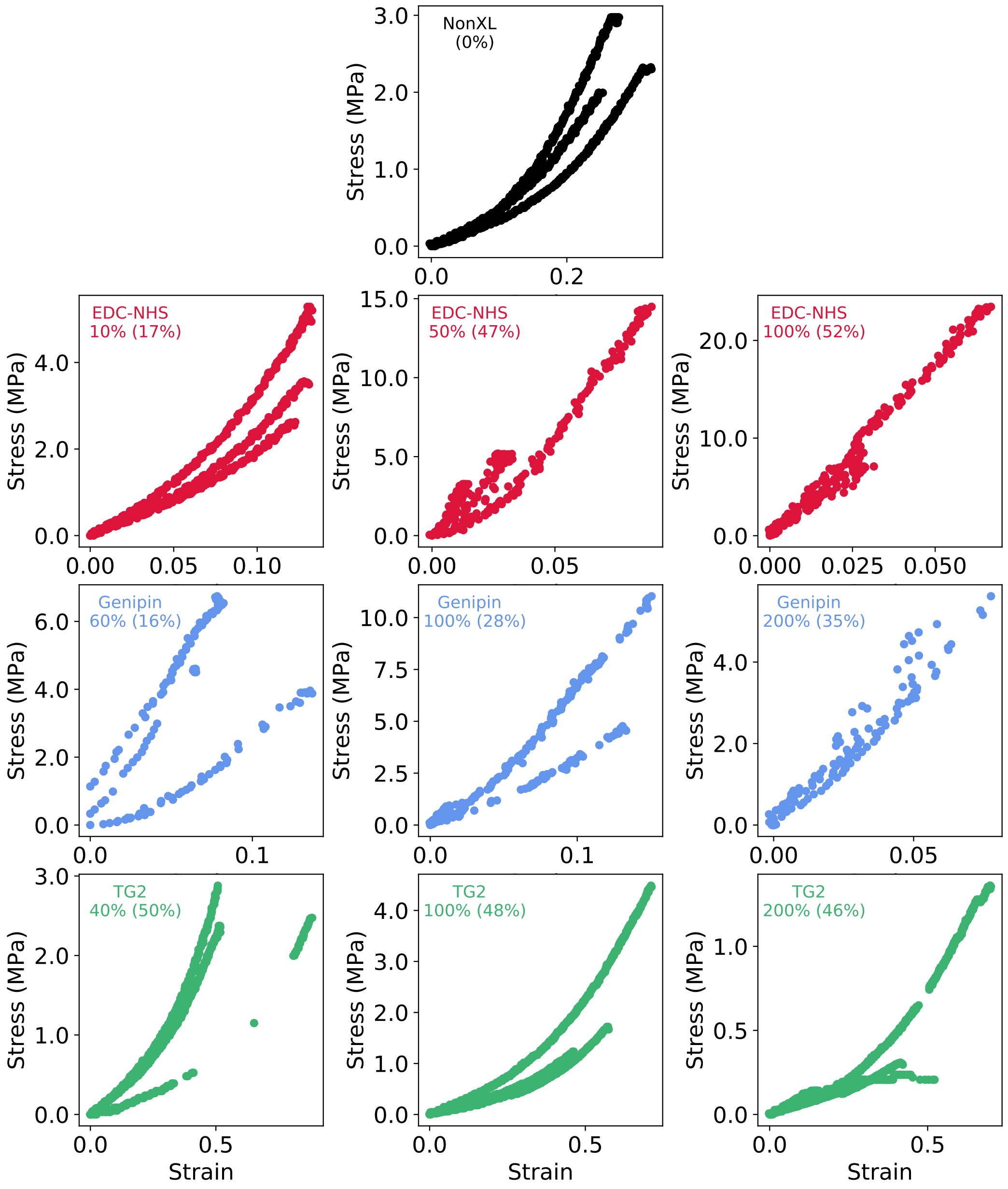

Figure 3: Representative stress strain curves of the non-crosslinked, EDC-NHS, genipin and TG2-treated collagen films. The curves possess four regions of interest: an initial toe region, linear-elastic regime, plastic deformation and finally catastrophic failure. Increasing EDC-NHS and genipin crosslinking increases film strength and modulus, while decreasing plasticity and strain to failure. TG2-treatment increases the ductility of the films while decreasing strength and modulus.

4. Quantitative cell characterisation

Cell area, perimeter, circularity, aspect ratio and roundness were subsequently measured for 10 cells in total across independent experiments performed in quadruplicate.

$$\text{Circularity} = \frac{4\pi \times \text{Area}}{\text{Perimeter}^2}$$

$$\text{Roundness} = \frac{4 \times \text{Area}}{\pi \times \text{Major Axis}^2}$$

Circularity and round are shape parameters that can help delineate distinct stages of cell spreading characterised by cell polarisation by considering the relative effects of perimeter, area and aspect ratio. Roundness is a direct measure of the cell polarisation and aspect ratio, whereas circularity is an indirect measure of the peripheral smoothness by including the effect of contact perimeter.

	Area / µm^2	Perimeter / µm	Circularity	Aspect Ratio	Round
NonXL (0%)	2000±1000	400 ± 200	0.21 ± 0.09	1.9 ± 0.5	0.6 ± 0.2
EDC-NHS 10% (17%)	1900 ± 900	400 ± 200	0.2 ± 0.1	1.7 ± 0.3	0.6 ± 0.1
EDC-NHS 50% (47%)	1700 ± 400	400 ± 200	0.18 ± 0.08	1.3 ± 0.3	0.8 ± 0.1*
EDC-NHS 100% (52%)	1600 ± 900	300 ± 200	0.2 ± 0.2	1.4 ± 0.3	0.8 ± 0.1*
Genipin 60% (16%)	2100 ± 600	400 ± 100	0.17 ± 0.06	1.6 ± 0.4	0.7 ± 0.2
Genipin 100% (28%)	2400 ± 900	400 ± 100	0.2 ± 0.1	1.6 ± 0.3	0.8 ± 0.1*
Genipin 200% (35%)	2100 ± 800	400 ± 100	0.2 ± 0.1	1.6 ± 0.3	0.77±0.09*
TG2 40% (50%)	2100 ± 900	500 ± 100	0.13 ± 0.05	1.6 ± 0.4	0.71 ± 0.09
TG2 100% (48%)	2300 ± 900	500 ± 100	0.16 ± 0.09	1.6 ± 0.3	0.7 ± 0.1
TG2 200% (46%)	1700 ± 600	400 ± 100	0.17 ± 0.07	1.6 ± 0.4	0.77±0.09*

Table 1: Mean area, perimeter, circularity, aspect ratio and round values obtained from single cell spreading images. * indicates a statistically significant difference in mean cell spreading parameters from the non-crosslinked condition of collagen. Results are reported as means ± standard deviations of measurements.

The data reveal no significant differences in the mean cell area, cell perimeter or circularity of the cells when comparing cells on treated substrates to the non-crosslinked condition. However, a significant increase in cell roundness is seen at 50% and 100% EDC-NHS, 100% and 200% genipin, and 200% TG2 when compared with the non-crosslinked condition. Although values can vary significantly between cell types, fully spread fibroblasts have been reported to have an area of 1250 µm^2 as opposed to the 300 µm^2 surface area of unspread rounded fibroblasts.[2] In comparison with these values, the higher cell area for all conditions indicates that the cells are well-spread on all conditions of crosslinking, actively maximise their area of contact with the substrate. The cell perimeter has been reported to represent both the affinity of the cell for a given surface but also can reflect different stages of cell spreading.[2]

5. Live-Dead Imaging

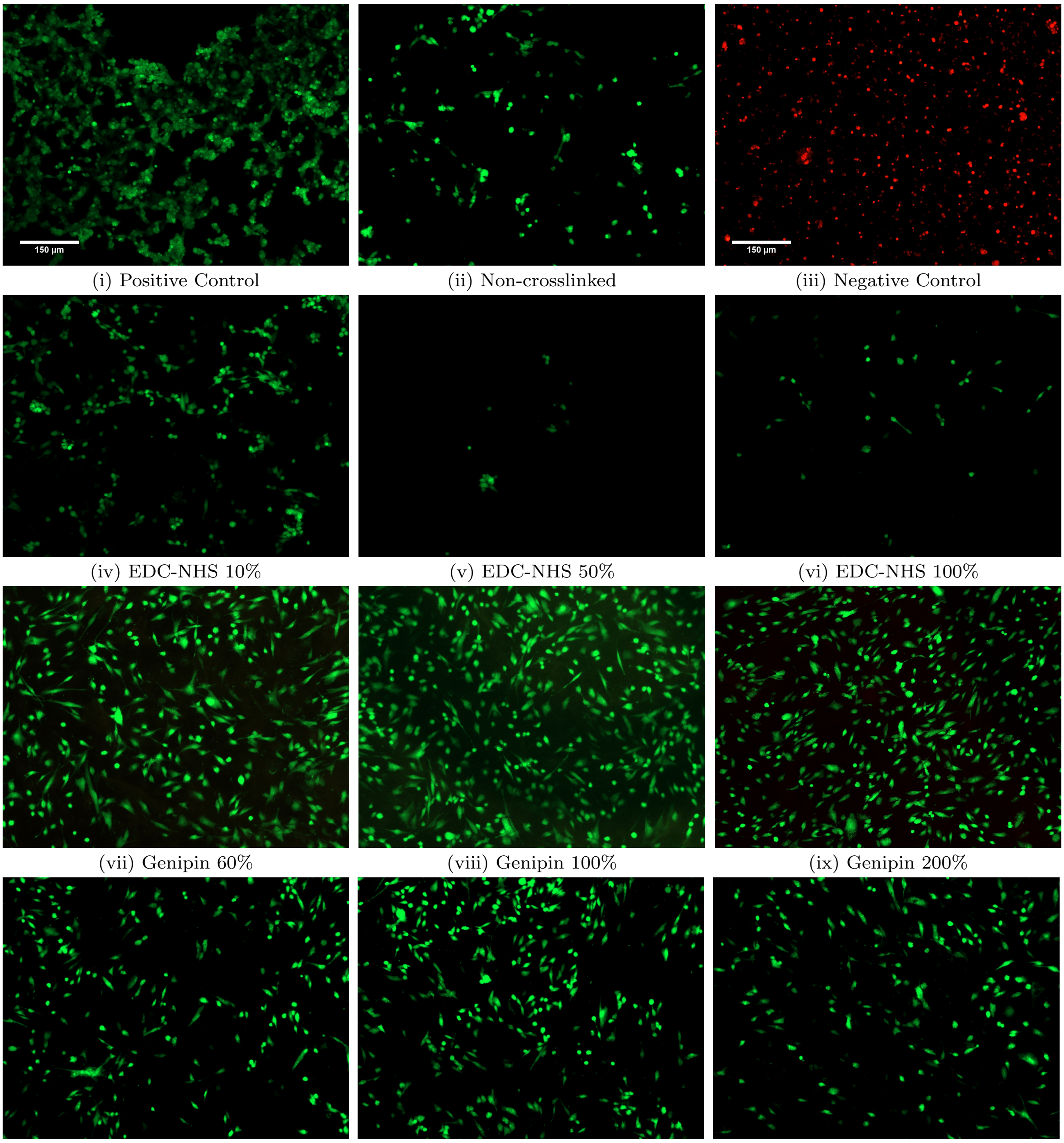

(i) Positive Control (ii) Non-crosslinked (iii) Negative Control

(iv) EDC-NHS 10% (v) EDC-NHS 50% (vi) EDC-NHS 100%

(vii) Genipin 60% (viii) Genipin 100% (ix) Genipin 200%

(x) TG2 40% (xi) TG2 100% (xii) TG2 200%

Figure 4: Cytotoxicity analysis of human dermal fibroblasts cultured on chemically crosslinked collagen films. Day 1 morphology of fibroblasts grown on tissue culture plastic was used as a positive control and fibroblasts treated for 20 minutes with 3% glutaraldehyde was used as a negative control. Red=EthD, green=Calcein.

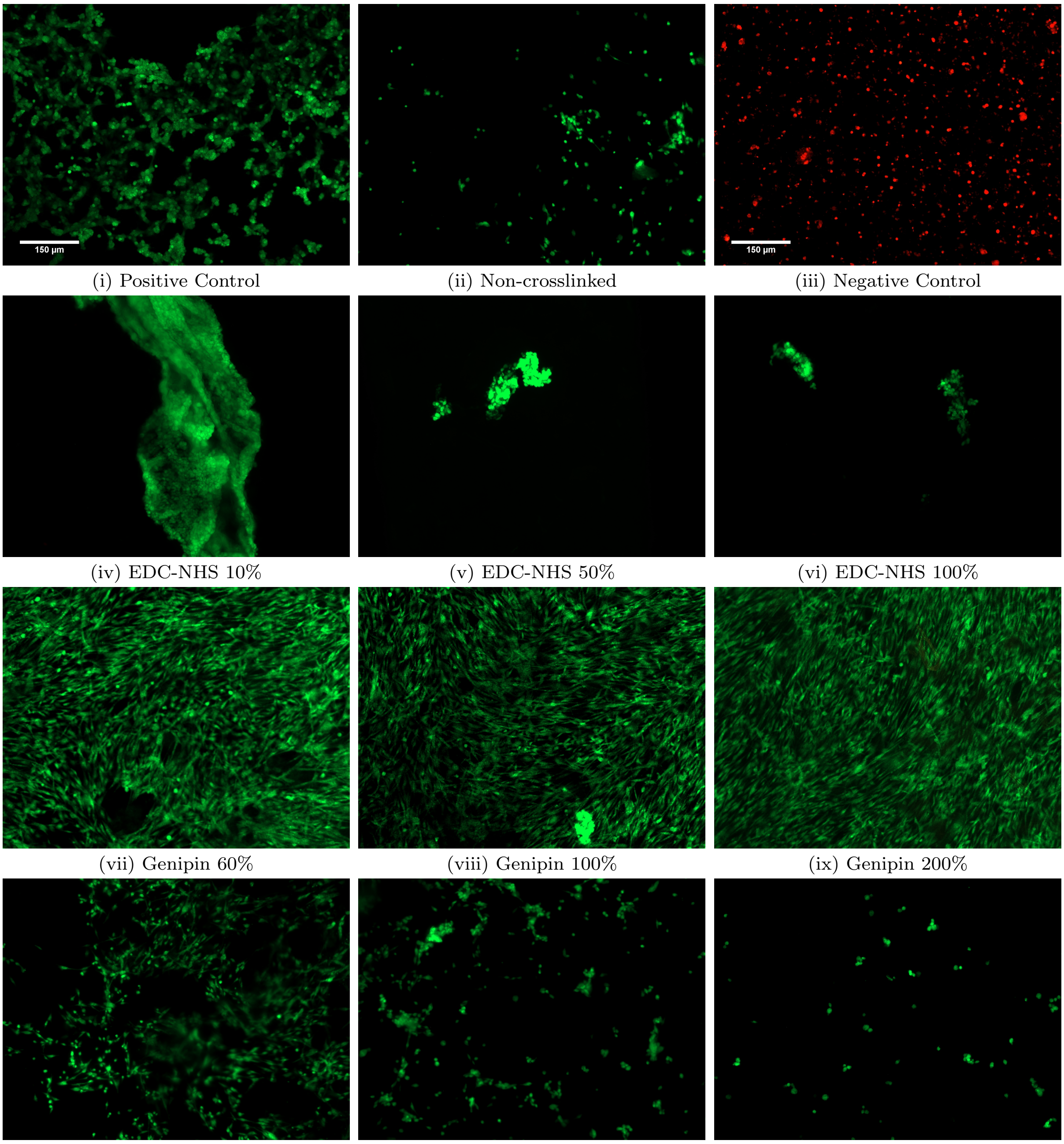

(i) Positive Control (ii) Non-crosslinked (iii) Negative Control
(iv) EDC-NHS 10% (v) EDC-NHS 50% (vi) EDC-NHS 100%
(vii) Genipin 60% (viii) Genipin 100% (ix) Genipin 200%
(x) TG2 40% (xi) TG2 100% (xii) TG2 200%

Figure 5: Cytotoxicity analysis of human dermal fibroblasts cultured on chemically crosslinked collagen films. Day 4 morphology of fibroblasts grown on tissue culture plastic was used as a positive control and fibroblasts treated for 20 minutes with 3% glutaraldehyde was used as a negative control. Red=EthD, green=Calcein.

6. Collagen I expression

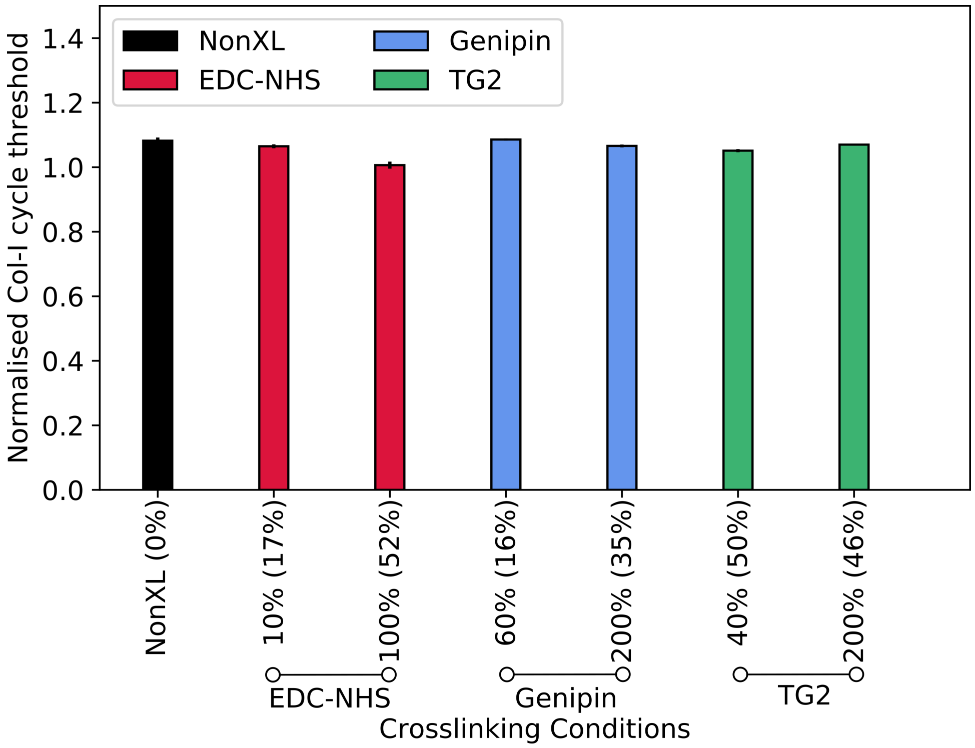

(a) GAPDH normalised cycle threshold for Col-I expression

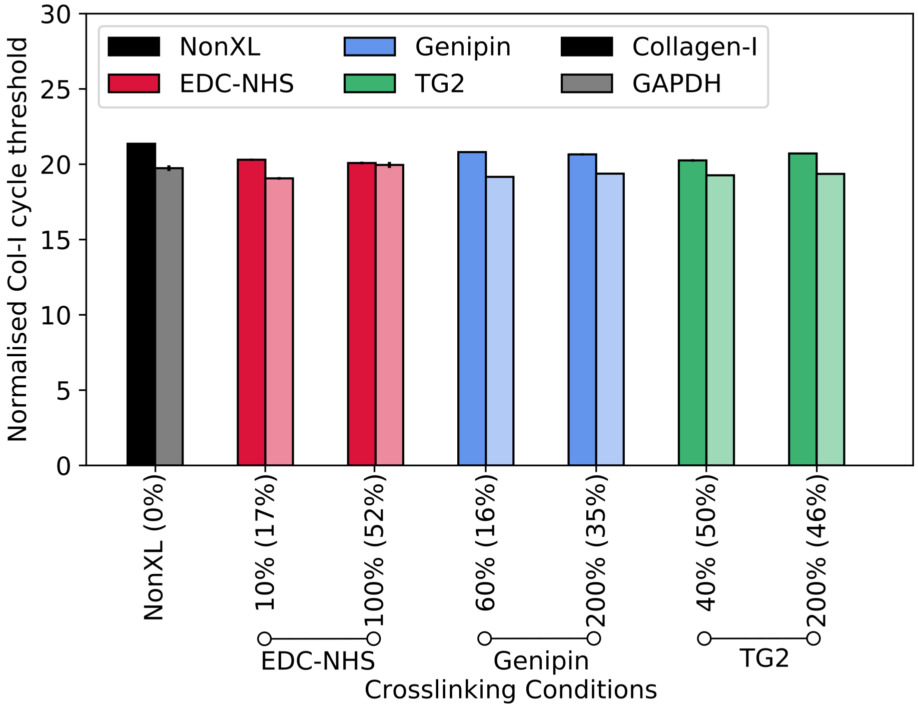

(b) Collagen-I and GAPDH expression

Figure 6: Expression of Collagen-I and GAPDH by HDFs seeded on untreated, EDC-NHS-, genipin- and TG2-treated collagen films.

The expression of Collagen-I and GAPDH by the human dermal fibroblasts seeded on untreated and EDC-NHS, genipin- and TG2-treated collagen films are shown in Figure 6, where similar levels of expression are observed for cells seeded on all untreated and treated substrates.

7. Human collagenase (MMP1) expression

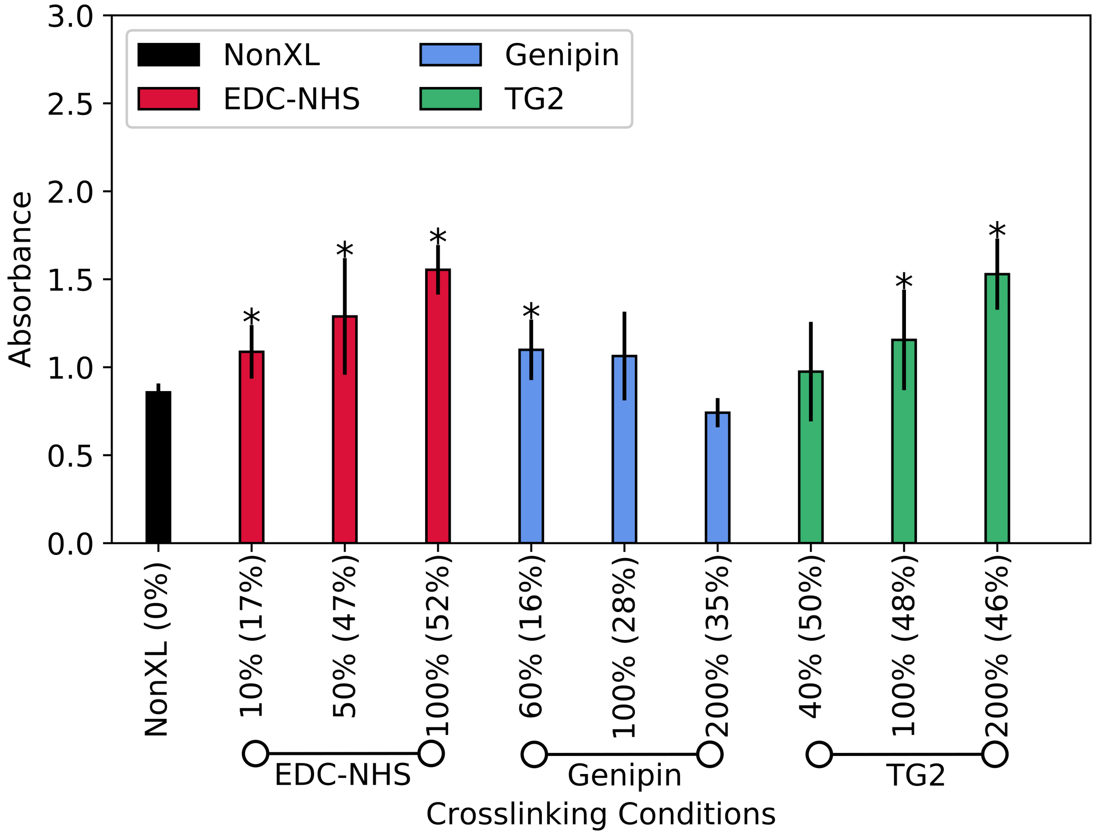

Figure 7: Expression of MMP-1 by HDFs seeded on untreated, EDC-NHS-, genipin- and TG2-treated collagen films. Samples exhibiting a significant difference from the non-crosslinked sample are noted with a *.

MMP1 expression seen in Figure 7 indicates an increase in the protease secretion on all EDC-NHS treated substrates, the low crosslinking concentratons of Genipin (40%) and the high crosslinking concentrations of TG2 (100 and 200%). Although this study cannot conclusively identify the mechanisms by which the different crosslinkers regulate MMP1 expression, we speculate that the deviation in the expected glutamic acid residues, arising both with EDC-NHS and TG2 treatment, but not with genipin treatment, may result in the upregulation of MMP1 by the human dermal fibroblasts.

8. Swelling

In general, the treatments were not observed to affect the material significantly as seen in Figure 8, with the exception of a statistically significant decrease in water uptake at the highest crosslinking concentrations of genipin and TG2. As a result, the low

swelling of the films when combined with their low thickness, little to no cell-infiltration is expected within the films and all cellular behaviour is likely to be limited to the surface of the films. It must also be noted that the trends observed here on the thin collagen films only reflect changes in hydrophilicity and are not comparable to 3D hydrogels or porous scaffolds where several networks for water infiltration are available. In such 3D constructs, other materials properties such as the mechanical strength will also play a role in the ability of the hydrogels to swell.

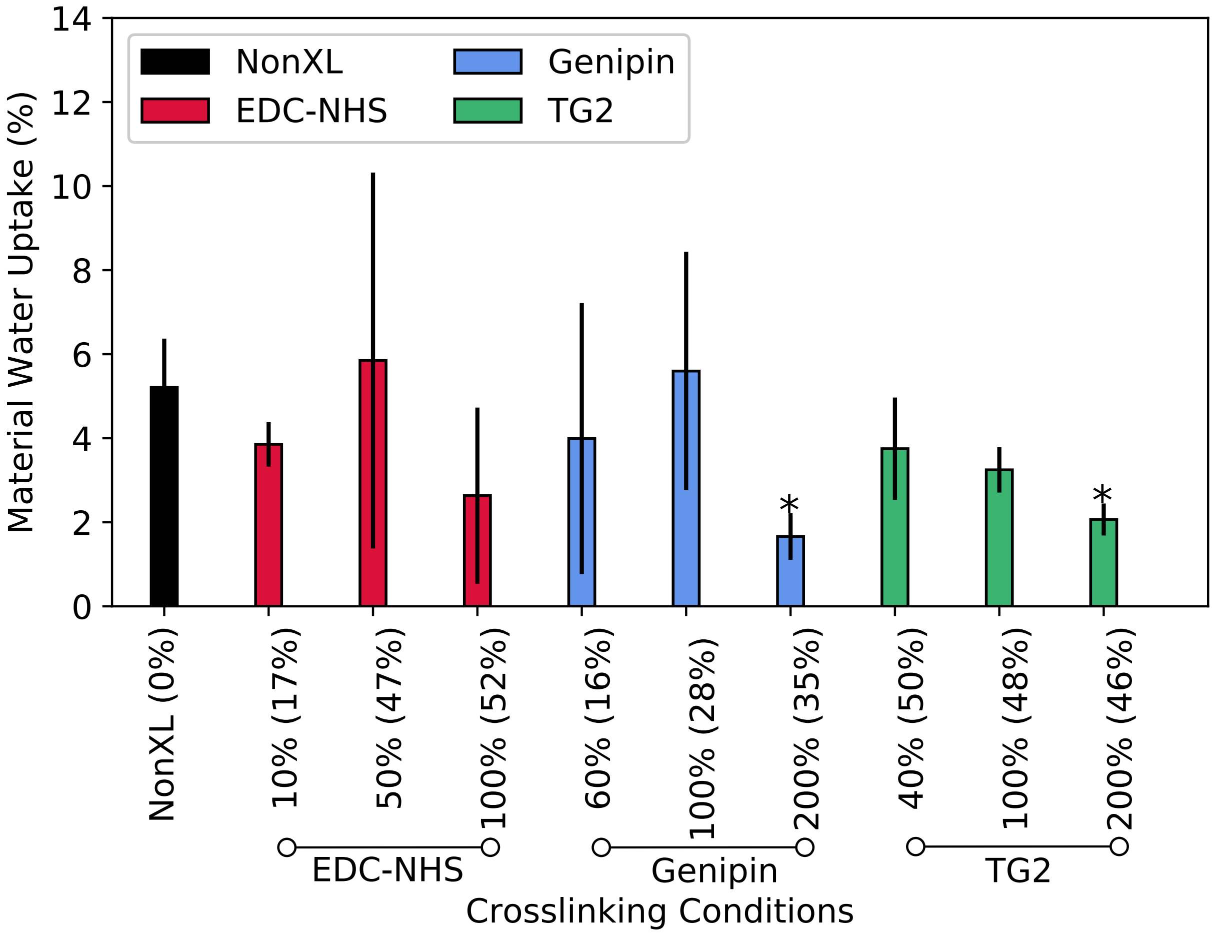

Figure 8: Water uptake of the non-crosslinked and treated collagen films after 24 hours of incubation in deionised water at 37 °C. Total material swelling is generally low, and significant differences are only observed at the highest crosslinking conditions of genipin and TG2. Samples exhibiting a significant difference from the non-crosslinked sample are noted with a *.

9. Photographs and Optical Micrographs

As seen in the films shown in Figure 9, all films are clear and colourless with the exception of genipin-treated films which are clear but blue in colour after crosslinking. In spite of the blue colour of genipin-crosslinked constructs, the crosslinker has been extensively investigated *in vivo* for its use in corneal implants.[3,4,5] No delamination or macroscopic shrinkage is observed on the films adhered to the well plates after 7 day cell culture on the films, indicating good stability of films during the cell culture period.

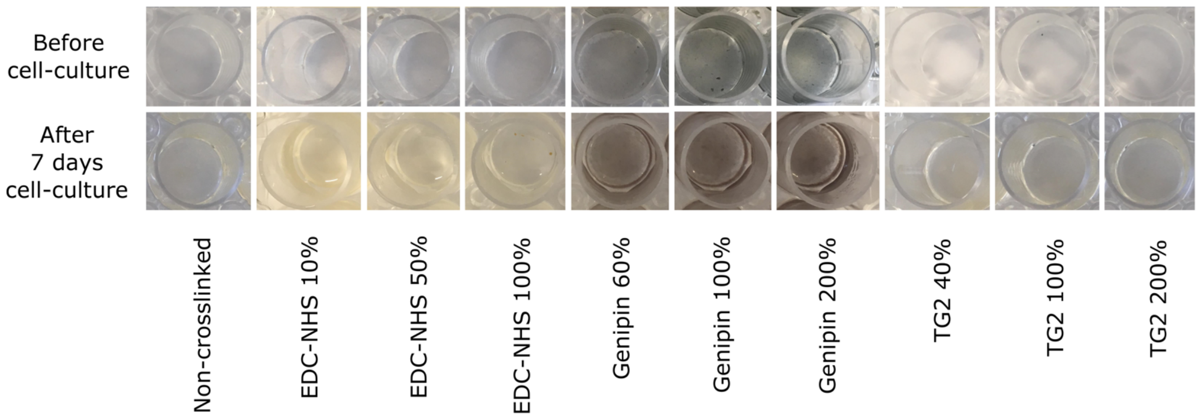

Figure 9: Images of cast collagen films on a 24 well plate after crosslinking, and following 7 days of cell culture.

Transmission phase contrast images of the films following 7 days of cell culture are also shown in Figure 10. At low crosslinking conditions (all TG2 and low EDC-NHS crosslinking), some shrinkage/ loss of material is observed around the edges of the films. Regardless, the central regions where cells were seeded and imaged are all observed to be well-adhered and unaffected over the 7-day cell culture period. Images were obtained using the Axiocam 503 Zeiss Fluorescence Microscope.

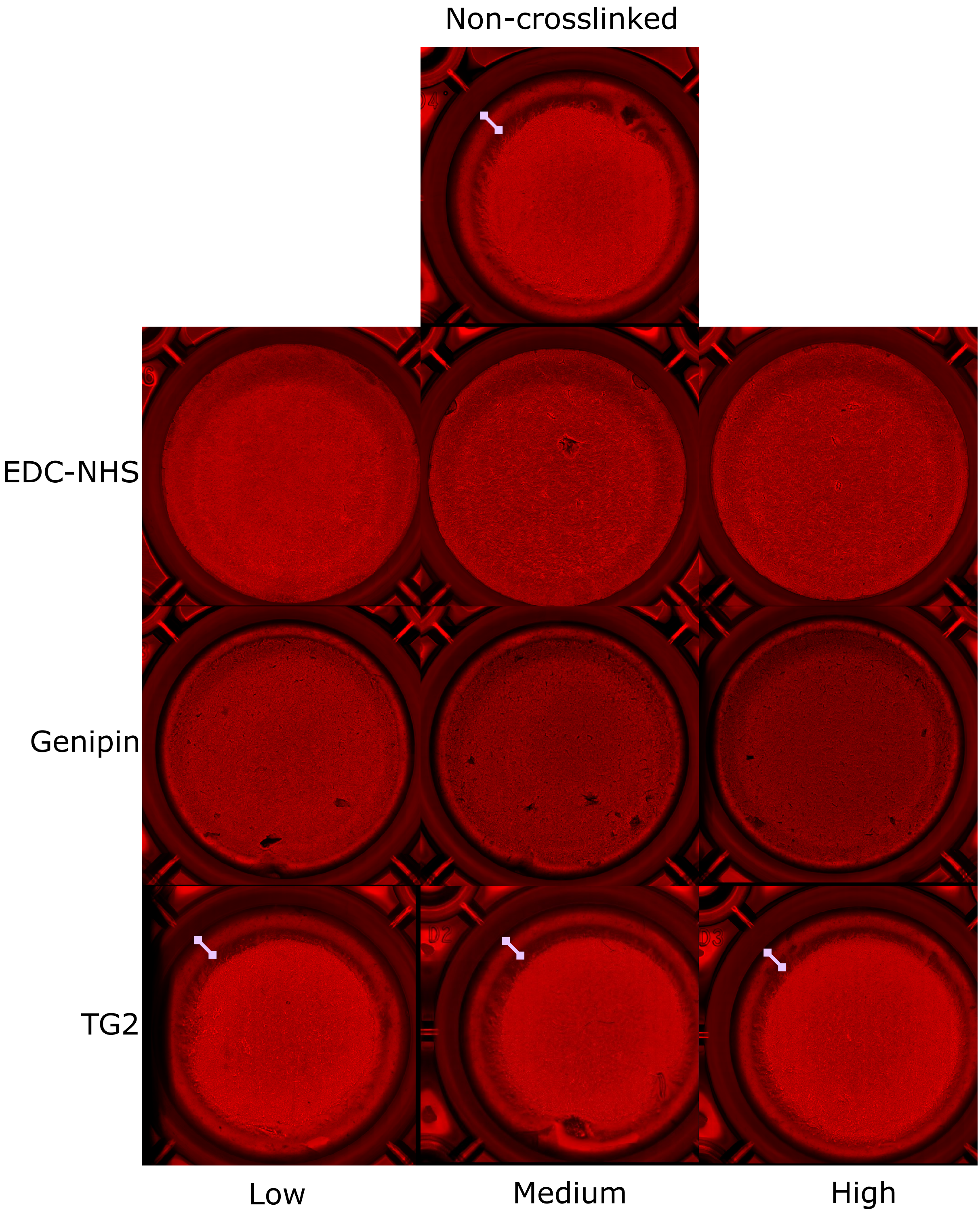

Figure 10: Phase contrast micrographs of untreated, EDC-NHS, genipin- and TG2-treated films. All films are well-adhered to the well-plates although some shrinkage and loss of material is observed at the low crosslinking conditions of EDC-NHS, the non-crosslinked conditions as well as all the TG2-treated samples, around the periphery of the films as indicated by the lilac lines.

References

[1] A. Terzi, E. Storelli, S. Bettini, T. Sibillano, D. Altamura, L. Salvatore, M. Madaghiele, A. Romano, D. Siliqi, M. Ladisa, *et al.*, “Effects of processing on structural, mechanical and biological properties of collagen-based substrates for regenerative medicine,” *Scientific reports*, vol. 8, no. 1, pp. 1–13, 2018.

[2] M. Brugmans, J.-J. Cassiman, L. Vanderheydt, A. J. Oosterlinck, R. Vlietinck, and H. Van Den Berghe, “Quantification of the degree of cell spreading of human fibroblasts by semi-automated analysis of the cell perimeter,” *Cytometry Part A*, vol. 3, no. 4, pp. 262–268, 1983.

[3] M. Y. Avila, M. Narvaez, and J. P. Castañeda, “Effects of genipin corneal crosslinking in rabbit corneas,” *Journal of Cataract & Refractive Surgery*, vol. 42, no. 7, pp. 1073–1077, 2016.

[4] M. Y. Avila, V. A. Gerena, and J. L. Navia, “Corneal crosslinking with genipin, comparison with uv-riboflavin in ex-vivo model,” *Molecular vision*, vol. 18, p. 1068, 2012.

[5] A. M. Gharaibeh, V. Saez, N. Garcia, L. Bataille, and J. L. Alió, “Optimizing genipin concentration for corneal collagen cross-linking: An ex vivo study,” *Ophthalmic research*, vol. 60, no. 2, pp. 100–108, 2018.
